# Near-infrared MINFLUX imaging enabled by suppression of fluorophore blinking

**DOI:** 10.1126/sciadv.adw3149

**Published:** 2025-12-05

**Authors:** Chinmaya V. Srambickal, Hanie M. Esmaeeli, Joachim Piguet, Lenny Reinkensmeier, René Siegmund, Ana Agostinho, Mark Bates, Alexander Egner, Jerker Widengren

**Affiliations:** ^1^ Experimental Biomolecular Physics, Bio-Opto-Nano Unit, Department of Applied Physics, KTH, Royal Institute of Technology, SE-10691 Stockholm, Sweden.; ^2^ Department of Optical Nanoscopy, Institute for Nanophotonics, D-37077 Göttingen, Germany.; ^3^ Science for Life Laboratory, Department of Applied Physics, Royal Institute of Technology, Solna 17165, Sweden.

## Abstract

MINimal photon FLUXes (MINFLUX) offers nanometer localization precision, with lower fluorophore requirements than for other super-resolution microscopy (SRM) techniques. Nonetheless, low localization probabilities hamper its application, and use of less bright and photostable fluorophores, including near-infrared (NIR) fluorophores has been difficult to realize. Here, we devised strategies overcoming these limitations. We systematically studied the blinking properties of far-red and NIR cyanine fluorophores, followed by simulations of MINFLUX localizations, over typical time scales (microsecond to 10 milliseconds), sample and excitation conditions for MINFLUX imaging. We identified fluorophore blinking via photoisomerization and photoreduction as the main cause of localization errors, and that use of balanced redox buffers and repetitive excitation beam scans can suppress such errors. Implementing these strategies, we could demonstrate NIR-MINFLUX imaging with nanometer localization precision, thereby also presenting an overall strategy to design optimal sample and excitation conditions, for MINFLUX imaging and for SRM in general.

## Teaser

Characterization and control of overlooked fluorophore blinking opens avenues for MINFLUX nanoscopy.

## INTRODUCTION

Following the development of far-field super-resolution microscopy (SRM), including coordinate-targeted stimulated emission depletion (STED) (*1*, *2*) and single-molecule localization microscopy (SMLM) (*3*, *4*), MINimal photon FLUXes (MINFLUX) (*5*, *6*) represents a next-generation SRM technique taking fluorophore localization precision to new levels, with less fluorescence photons required for the localization. The more relaxed requirements on molecular brightness and photostability in MINFLUX can open for the use of new categories of fluorophores, in particular near-infrared (NIR) fluorophores, which due to limited photon budgets have found a quite minor use in SRM this far. In MINFLUX, the fluorophores to be localized are first switched individually, typically on a slower time scale of milliseconds or longer, like in SMLM. However, while SMLM localizes individual, spatially separable, emitting fluorophores from the centroids of their emitted fluorescence as imaged by a camera, the MINFLUX concept uses a movable excitation beam with an intensity minimum, e.g., a donut beam as used in STED imaging. The coordinates of individual fluorophores can then be inferred from the detected fluorescence photon numbers relative to one another at the different beam positions, forming a so-called targeted coordinate pattern (TCP) around the fluorophore to be localized. The excitation intensity experienced by a fluorophore will be weaker the closer it is to the minimum of the excitation beam for a laser beam position within a TCP. Ideally, if the excitation minimum is zero, the absence of fluorescence emission can provide a signature that the excitation zero coincides spatially with the fluorophore. MINFLUX, acknowledged as the most efficient known way of using fluorescence photons for localizations (*7*), thus opens for the use of emitters with lower photon budgets (lower molecular brightness and photostability), but on the other hand also puts other requirements in the forefront, such as low background levels (*5*). Moreover, markedly lowered localization probabilities of fluorophores have been experienced in MINFLUX imaging experiments, compared to in SMLM imaging (*7*, *8*). The underlying mechanisms have this far remained unresolved, but are addressed in this work.

In recent years, fluorescence imaging in the NIR range has received increased attention and comes with several advantages, including reduced scattering, lower absorption, lower autofluorescence, lower phototoxicity in the sample, as well as deeper penetration depths (*9*–*11*). Given the relatively strict requirements on fluorophore photostability and brightness in SRM (*12*, *13*), and since organic fluorophores generally display lower fluorescence brightness and photostability the more red-shifted their excitation and emission spectra, NIR fluorophores have hitherto found limited use in SRM. However, in MINFLUX imaging the generally lower photon budgets of these fluorophores should be better tolerated. Moreover, excitation and emission in the NIR provide prerequisites for reaching lower background levels (e.g., from autofluorescence) and offer additional spectral windows for simultaneous SRM imaging over multiple, spectrally separated color channels.

In the red spectral range, pentamethine cyanines such as Alexa Fluor 647 (AF647) have become fluorophores-of-choice for SMLM, in particular for stochastic optical reconstruction microscopy (STORM) (*4*, *14*), combining the necessary fluorophore brightness and photostability with appropriate slow switching properties (number of switching cycles, ON-OFF duty cycle) (*15*). These properties and its usefulness for STORM imaging have also qualified AF647 as one of the first fluorophores to be established for MINFLUX imaging (*5*, *6*). For clarity, with “switching” we mean any reversible ON-OFF process imposed on the fluorophores to assure that only one fluorophore at a time is ON (in an excitable and emissive state) within a diffraction-limited volume. We refer to other fast ON-OFF transitions of the fluorophores as “blinking.” In the NIR, heptamethine cyanines by far represent the most commonly used fluorophore category for bioimaging and can be expected to show similar slow switching properties as AF647 and other pentamethine cyanines (*15*). While they typically lack the brightness and photostability to make them attractive for STORM imaging, it can be argued that they may still qualify for MINFLUX imaging, for which the photon budget requirements are lower. However, even in the visible range, using fluorophores with better photon budgets, it has been noted that current MINFLUX implementations tend to detect fewer fluorophores, i.e., to show lower probabilities of localizing fluorophore-labeled target molecules, compared to camera-based (wide-field) SMLM techniques (*7*, *8*). It is unlikely that these lowered probabilities are related to the MINFLUX concept itself (*16*), but rather to properties of the fluorophores used. In the MINFLUX localization procedure, the fluorophore to be localized must first switch in a slow (milliseconds to seconds) timescale, and with a duty cycle low enough (typically 10^−3^ to 10^−4^) to allow individual, well-separated fluorophores to be targeted in the localization. This requirement is shared with SMLM (*15*) and should thus not lead to a lowered localization probability in MINFLUX compared to in SMLM. In the next MINFLUX localization step(s), however, with a donut-shaped excitation beam scanned around the emissive fluorophore in a predefined TCP, it is typically assumed that the fluorescence signal is linearly proportional to the excitation intensity experienced by the fluorophore. If the fluorophore undergoes faster blinking between different scanning positions of the donut beam, however, this linearity assumption is no longer fully valid, and the ability to infer the fluorophore localization from its emitted fluorescence at each of the scanning positions may be compromised. Such effects can in fact be a major reason for the recently reported, lowered localization probabilities experienced in MINFLUX imaging (*7*, *8*). Accordingly, given the similarities in blinking/switching properties between NIR heptamethine and far-red pentamethine cyanines such as AF647, blinking may also be a major complication for the use of heptamethine cyanines, and possibly a major hurdle needed to overcome to make NIR-MINFLUX imaging possible. While effects of fluorophore blinking in MINFLUX measurements has been brought up as a possible complication (*17*), a quantitative analysis and verification of such effects has not been reported to our knowledge. Such investigation is relevant for MINFLUX imaging, but also for other SRM techniques incorporating time-modulated illumination patterns in the localization (*18*–*20*), and can provide an understanding, whereby imaging strategies can be modified to minimize or eliminate localization errors. Consequently, here, we performed systematic studies of far-red and NIR cyanine fluorophore blinking with subsequent simulations of MINFLUX localizations. This allowed us to design optimal sample and excitation conditions and then to demonstrate NIR-MINFLUX imaging in practice. More generally, this work can thereby also open for a broader use of NIR fluorophores in MINFLUX and related SRM studies.

## RESULTS

To understand how and to what extent fluorophore blinking can affect localization precisions and probabilities in MINFLUX imaging, and to develop possible remedy strategies, we systematically studied the blinking properties of the pentamethine cyanine AF647, over a time scale spanning several orders of magnitude (microsecond to 10 ms), and under labeling, sample, and excitation conditions relevant for MINFLUX imaging. Similarly, we also studied three heptamethine cyanine fluorophores; Dylight 755 (DL755), AF750, and CF750, as possible candidate fluorophores for MINFLUX imaging in the NIR, displaying similar (STORM compatible) slow switching properties as AF647 (*15*). From the acquired data, photodynamic models with rate parameters were determined, based on which we then performed simulations of the fluorophore photodynamics and blinking behavior, under representative MINFLUX excitation beam scans and sample conditions. From these simulations, we could identify major experimental conditions and fluorophore blinking properties compromising the localization precision and probability, allowing us to formulate strategies to minimize these effects. Last, we then applied these strategies in practice, demonstrating NIR MINFLUX imaging with nanometer localization precision.

### Photophysical characterization

We applied transient-state (TRAST) spectroscopy, offering a robust approach to monitor fluorophore blinking kinetics over a μs to 10 ms time range (*21*, *22*), performed on a customized wide-field microscope setup, with laser excitation at 640 nm (for AF647) or 750 nm (for DL755, AF750, and CF750) (see Materials and Methods). By so-called TRAST curves, plotting the time-averaged fluorescence intensity, ${\langle F_{\text{exc}}\left(w\right)\rangle }_{\text{norm}}$ , generated by rectangular excitation pulse trains with different pulse widths, *w*, characteristic relaxations on a microsecond to millisecond timescale were recorded in the fluorophore samples. These relaxations reflect buildup of photoinduced non- or weakly emissive states in the fluorophores, generated following onset of the excitation pulse (see text S1 and eqs. S1 to S4). From the relaxations recorded in the TRAST curves, we established a model for the underlying state transitions in the fluorophores and determined their transition rates under different excitation and sample conditions relevant for MINFLUX imaging and SMLM.

**Fig. 1.**
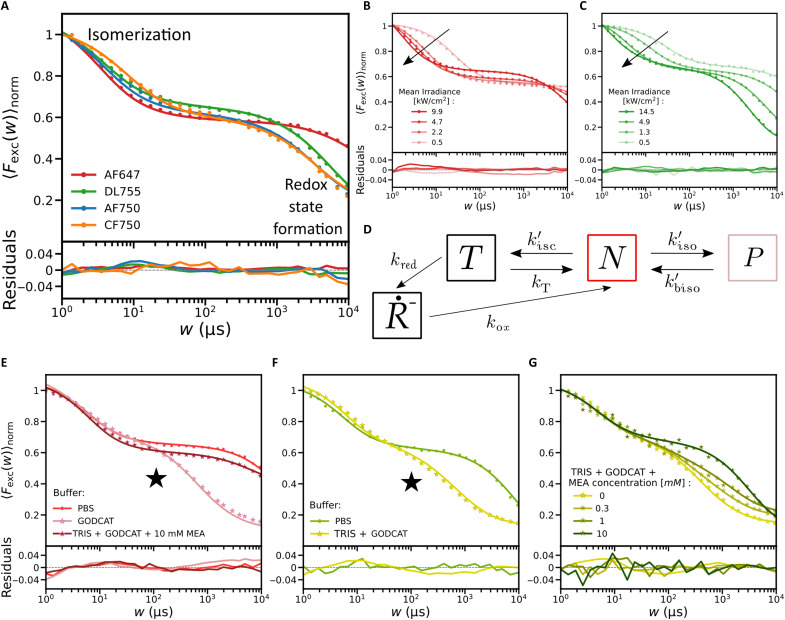
TRAST data and photophysical model. Experimental TRAST curves (dots), with corresponding fitted curves (lines), and fitting residuals plotted below. If not stated otherwise, the TRAST curves were recorded in air-saturated PBS solution, with excitation at 750 nm for the NIR dyes and at 638 nm for AF647. (**A**) TRAST curves recorded from free NIR fluorophores (excitation intensity, $I_{\text{exc}}$ = 4.9 kW/cm^2^) and from AF647 ( $I_{\text{exc}}$ = 4.7 kW/cm^2^) in an air-saturated PBS buffer solution (11.8 mM, pH 7.4). (**B**) Excitation intensity dependence for AF647. Arrow indicates increasing intensities. (**C**) Excitation intensity dependence for DL755. Arrow indicates increasing intensities. (**D**) Photophysical model, containing an all-trans (N), photoisomerized (P), triplet (T), and photoreduced state ( ${\dot{R}}^{-}$ ), as described in the Results and used to fit the experimental TRAST curves. (**E**) TRAST curves recorded from AF647, in PBS, when in a deoxygenated buffer (tris + GODCAT) and upon addition of MEA (10 mM) ( $I_{\text{exc}}$ = 4.7 kW/cm^2^). Black star: triplet-state buildup. (**F**) Effects of deoxygenation (GODCAT) in DL755 ( $I_{\text{exc}}$ = 4.9 kW/cm^2^). Black star: triplet-state buildup. (**G**) TRAST curves recorded from DL755 in tris +GODCAT with different concentrations of MEA added ( $I_{\text{exc}}$ = 4.9 kW/cm^2^).

TRAST curves recorded from the NIR cyanines and AF647 all displayed two major dark-state relaxation processes (Fig. 1A). The faster relaxation (1 μs to 100 μs) showed a largely constant amplitude (30 to 40% for the different fluorophores), while its relaxation time decreased with higher excitation photon fluxes, $\Phi_{\text{exc}}$ (Fig. 1, B and C, and fig. S1). This $\Phi_{\text{exc}}$ dependence is consistent with reversible excitation-driven isomerization between an emissive all-trans state, N, and one or several non- or weakly emitting photoisomerized states, P, as previously observed in fluorescence correlation spectroscopy (FCS) (*23*, *24*) and TRAST experiments (*25*, *26*). For the slower relaxation (1 to 10 ms), we observed an increase in its amplitude and a faster decay with higher $\Phi_{\text{exc}}$ (Fig. 1, B and C, and fig. S1), in agreement with formation of a photoreduced state, ${\dot{R}}^{-}$ , with a not excitation-driven recovery. For many cyanine fluorophores photoreduction is a major dark-state transition, reported to take place from the triplet state, T (*27*, *28*). In air-saturated buffer solutions, however, we did not observe any substantial buildup of the triplet state itself (Fig. 1, A to C), also not at higher $\Phi_{\text{exc}}$ . This is consistent with previous FCS and TRAST studies (*23*, *25*, *26*), reflecting efficient quenching of T by molecular oxygen, the relatively short fluorescence lifetimes in these fluorophores (text S2 and fig. S2) and that intersystem crossing is outcompeted by photoisomerization.

Notably, the TRAST curves in Fig. 1, A to C indicate that prominent dark-state transitions take place in the fluorophores, over a time range (1 μs to 10 ms) over which also MINFLUX beam localization procedures typically operate. To describe these relaxations and their effects on MINFLUX localizations, we assigned a four-state photophysical model (Fig. 1D) and a fluorophore state vector $\bar{A}\left(t\right)={\left[\left[N\right]\left(t\right),\left[P\right]\left(t\right),\left[T\right]\left(t\right),\left[{\dot{R}}^{-}\right]\left(t\right)\right]}^{T}$ representing the population probabilities of the N, P, T, and ${\dot{R}}^{-}$ states (see text S3 for details). In absence of excitation, polymethine cyanine fluorophores typically exist in their all-trans state (*28*). For fluorophores subject to constant $\Phi_{\text{exc}}$ starting at time *t* = 0, we can then set $\bar{A}\left(0\right)={\left[1,0,0,0\right]}^{T}$  as initial condition. Relaxations in the generated fluorescence intensity, F(t) , as observed in TRAST curves (see text S4 and eqs. S14 to S16), can then be attributed to how [*N*] and [*P*] evolves within rectangular excitation pulses with increasingly longer pulse durations

$${\langle F_{\text{exc}}\left(w\right)\rangle }_{\text{norm}}=\frac{1}{w}\int_{t=0}^{w}F\left(t\right)/F\left(0\right)\mathrm{dt}=\frac{1}{w}\int_{t=0}^{w}\left[\left[N\right]\left(t\right)+Q\cdot \left[P\right]\left(t\right)\right]\mathrm{dt}$$ (1)

Here, *Q* denotes the relative brightness factor of P versus N (text S3 and eq. S13), attributed to a weak, red-shifted emission from P observed in the TRAST curves (see text S5 and fig. S3).

To further evaluate the model of Fig. 1D, fitting of photophysical rate parameters was performed as previously described (*25*, *26*, *29*–*31*), based on Eq. 1 (see text S4 for further details). For each of the fluorophore samples, global fitting of rate parameters to multiple TRAST curves, recorded at different $\Phi_{\text{exc}}$ could well reproduce the experimental data (Fig. 1, B and C and fig. S1). The fitted parameter values are listed in table S1.

Next, we studied how the photophysical transitions were affected by the sample conditions used for STORM and MINFLUX imaging. Compared to free fluorophores, conjugation to antibodies resulted in longer isomerization relaxation times (fig. S4), with both isomerization and back-isomerization rates lowered (table S1). STORM imaging buffers typically contain an enzyme-based oxygen scavenger system (GODCAT, see Materials and Methods), together with a thiol-based reductant, such as mercaptoethylamine (MEA). As expected, deoxygenation by GODCAT led to a prominent increase in the triplet-state (T) buildup, while titration of MEA into the deoxygenated buffer resulted in depopulation of T into the ${\dot{R}}^{-}$ state (Fig. 1, E to G, and fig. S5). Notably, however, the ${\dot{R}}^{-}$ relaxation occured on a much (more than an order of magnitude) faster time scale than in STORM experiments (*15*). For cyanines in their triplet state, thiols can promote photoinduced electron transfer, with subsequent triplet-to-singlet intersystem crossing in geminate cyanine-thiol radical pairs. These radical pairs can then undergo transitions along two separate paths: Either radical pair dissociation and back-electron transfer into emissive singlet-state fluorophores may take place, or more long-lived, nonemissive cyanine-thiol adducts are formed (*32*). The latter forms the basis for the slow switching required for STORM measurements (*14*, *33*), while our interpretation is that the TRAST measurements reflect cyanine-thiol radical pairs in which the cyanines undergo a faster back-electron transfer upon radical pair dissociation (*32*). In this case, there is no subsequent adduct formation, resulting in a much more short-lived ${\dot{R}}^{-}$ state. The experimental TRAST curves recorded from samples with GODCAT and MEA could be well reproduced by rate parameter fitting, using the same photophysical model as for the free fluorophores (Fig. 1D). The fitted rate parameters (table S1) reflect the experimental observations (Fig. 1G), with MEA promoting triplet-state deactivation in the GODCAT samples by enhancing the triplet decay rate (*k_T_*) and its transition rate (*k*_red_) to ${\dot{R}}^{-}$.

Overall, from the TRAST experiments, we find that dark or dim states are generated to a substantial extent for all fluorophores, over a broad range of time scales. While switching into long-lived dye-thiol adducts is orders of magnitude slower, the dark-state transitions observed in the TRAST experiments (including more short-lived, nonadducted ${\dot{R}}^{-}$ state fluorophores) can readily fall within typical operation times of MINFLUX localization procedures and their TCP iterations (typically tens of microseconds up to milliseconds to complete a TCP). These transitions may thus challenge the linear relationship assumed in MINFLUX experiments, between the local $\Phi_{\text{exc}}$ experienced by a fluorophore and its fluorescence photon emission rate.

### Simulations

**Fig. 2.**
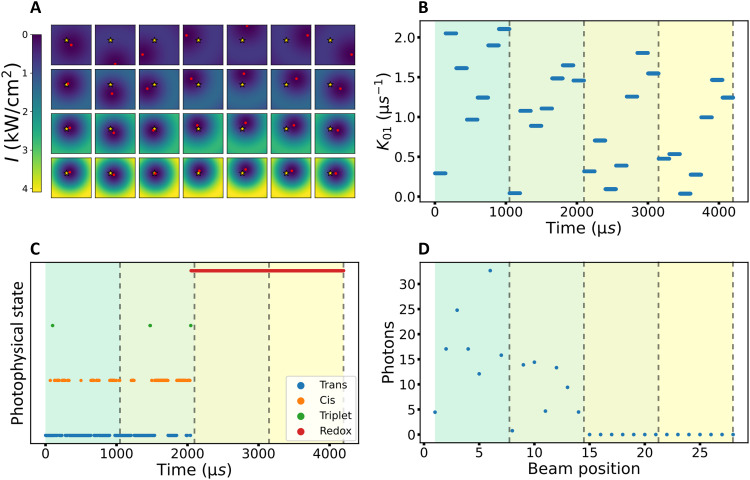
Simulation of a representative MINFLUX localization. (**A**) Illustration of the fluorophore position [golden star, in this example located at (−35 nm, 35 nm) with respect to the TCP center in the first iteration] and the different beam positions (beam centers marked with red dots) over the different TCP iterations (corresponding to each row), with the excitation intensity increasing and the diameter of the TCP (*L*) decreasing from one TCP iteration to the next. (**B**) The excitation rate ( $k_{01}$ ) experienced at the fluorophore position over time as the beams are moved in the four TCP iterations. (**C**) Photophysical state evolution showing the fluorophore undergoing transitions within the time of the localization between the bright trans isomer state (N, blue), the dark cis isomer (P, orange), triplet (T, green), and the likewise dark long-lived redox ( ${\dot{R}}^{-}$ , red) state. (**D**) The integrated photons collected from the fluorophore for each of the beam positions shown in (A). The different colored regions in (B), (C), and (D) indicate the different TCP iterations. The dashed lines indicate the end of each iteration.

To assess the effects of fluorophore blinking in MINFLUX localizations more in detail, we performed simulations for a fluorophore, undergoing blinking transitions as determined in the TRAST experiments above, and subject to a basic, representative two-dimensional localization algorithm used in MINFLUX imaging (*5*, *34*) (Fig. 2A, further details in text S6). A donut-shaped excitation beam was translated within a plane in a hexagonal TCP around the fluorophore located at position, ${\bar{r}}_{m}$ (Fig. 2A, gold star). In each iteration, the donut beam center (Fig. 2A, red dots) was first parked in the center of the hexagonal TCP and then at each of its six vertices, with the same beam dwell time, *t*_dwell_, at each of the positions. Within each iteration (represented by rows in Fig. 2A), fluorescence photon counts, $\bar{n}=\left\{n_{0},n_{1},n_{2},n_{3},n_{4},n_{5},n_{6}\right\}$ , generated from the fluorophore by the excitation beam in its seven beam positions relative to ${\bar{r}}_{m}$ were calculated as an integral over *t*_dwell_ for each beam position of the TCP. The calculations were based on the photophysical model (Fig. 1D) and transition rates (table S1) as determined for the fluorophore, with the all-trans state (N) as the only emissive state, and based on its corresponding excitation cross section, $\sigma_{N}$ , and fluorescence lifetime (text S2). We then applied a maximum likelihood estimator (MLE) algorithm, as applied in MINFLUX (*5*), to estimate the fluorophore position, ${\bar{r}}_{m}^{\text{MLE}}$ , best in agreement with $\bar{n}$ . The TCP center is then relocated to ${\bar{r}}_{m}^{\text{MLE}}$ and the localization procedure thereafter repeated in three more iterations, with the diameter, *L*, of the TCP pattern reduced in each iteration step, and the excitation power increased (illustrated in the three lower rows of Fig. 2A, further details in text S6).

In the simulations, the purpose was to analyze how blinking kinetics in the fluorophores affects the localization. We therefore did not include background contributions from other fluorophores in the sample, and also not detector losses or any collection efficiency differences. To simulate the evolution of photophysical states on a single fluorophore level, as in actual MINFLUX imaging experiments, we applied a Markovian chain model (text S6 and fig. S6), based on the model in Fig. 1D and the transition rates as determined in the TRAST experiments (table S1). Since it is not possible to include all possible scenarios for the localization procedure in the simulations, we confined the simulations to iterations after prelocalization, with the fluorophore location, ${\bar{r}}_{m}$ , inside the TCP, and with a few representative starting excitation powers, fluorophore locations, and beam dimensions considered. This allowed us to capture major effects and features following from fluorophore blinking within a limited number of representative scenarios.

First, we simulated possible photophysical state evolution outcomes for a single fluorophore (DL755, labeled to an antibody and in a tris + GODCAT buffer with 10 mM MEA), located at ${\bar{r}}_{m}$ from the center of the first TCP as shown in Fig. 2A. A representative outcome is shown in Fig. 2 (B to D). Excitation rates, as experienced by the fluorophore, are calculated throughout the procedure, from the irradiance at ${\bar{r}}_{m}$ for the different beam positions within each TCP iteration (Fig. 2B). From the state evolution (Fig. 2C), the fluorescence photon counts, $\bar{n}$ , are then calculated in the simulations after each TCP iteration and for each beam position (Fig. 2D). In the outcome shown in Fig. 2C, the simulated fluorophore switches reversibly between its emissive, all-trans (N) and dark cis (P) state during the first two TCP iterations, and only occasionally goes into its triplet state (T). Toward the end of the second iteration, at around 2 ms, the fluorophore transits into its photoreduced state ( ${\dot{R}}^{-}$ ) from which the fluorophore does not relax back within the simulated time. The simulated state evolution for this single fluorophore largely reflects the transient state dynamics, as observed in the TRAST experiments on an ensemble level (Fig. 1C). Notably, in the simulated example, the calculated $\bar{n}$ after each TCP iteration (Fig. 2D) is far from linearly proportional to the corresponding excitation rates experienced by the fluorophore in each of the beam positions (Fig. 2B). This illustrates that fluorophore blinking properties of relevant fluorophores and under relevant experimental conditions for MINFLUX imaging can distort the calculation of $\bar{n}$ . A distorted $\bar{n}$ will affect the MLE estimation of ${\bar{r}}_{m}^{\mathrm{MLE}}$ for the next iteration, whereby errors due to fluorophore blinking can propagate and accumulate in the localization procedure. When averaged over many single molecules, on an ensemble level, the population kinetics of the photophysical states will be reproducible for a given set of parameters (as verified in our simulations, figs. S7 and S8). However, because of the stochastic nature of single molecules, blinking kinetics and state evolutions will vary from one individual fluorophore to another, even if subject to the same experimental conditions.

**Fig. 3.**
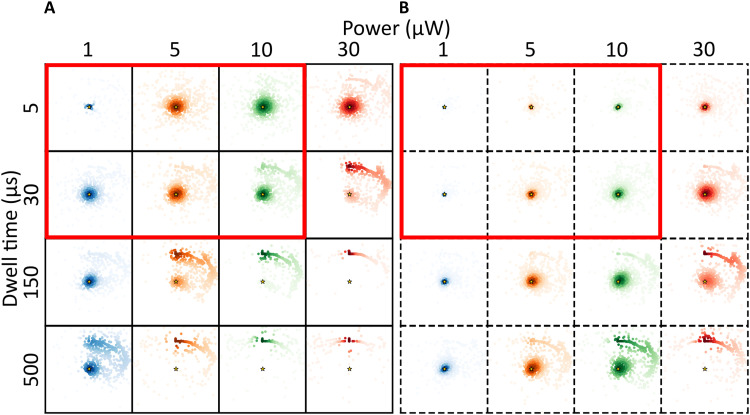
Maps of estimated locations from simulations of DL755. Colored dots represent simulated final estimated locations of a DL755 fluorophore at position (1 nm, 1 nm) (golden star) following an iterative MINFLUX localization. The figure shows simulated localization estimates (colored dots) for different excitation powers, different beam dwell times (times spent at each of the beam positions within the TCP in each of the iterations), and without (**A**) and with (**B**) pattern repeats (dashed boxes, pattern repeat of 5). The ROI (each box) has the dimensions −100 to +100 nm in the *x* and *y* directions. The difference in color transparency shows the density of estimated locations, calculated using a kernel density estimate (KDE) with a Gaussian kernel. Red-marked squares: Combinations of dwell time and excitation power whereby the total possible excitation time after a completed localization procedure, $t_{\text{total}}$ , will typically be smaller than the time needed for ${\dot{R}}^{-}$ buildup in the fluorophore (see Results for further details).

To assess how these stochastic features can affect the localization, we simulated 1000 localization outcomes for the same fluorophore (DL755) as simulated in Fig. 2, located at position (1 nm, 1 nm) near to the TCP center (0,0) at the start of the first iteration (Fig. 3). This round of simulations was repeated, applying different representative beam dwell times, *t*_dwell_, and starting laser powers, as used in experimental MINFLUX realizations. Figure 3A shows the resulting localization estimates ( ${\bar{r}}_{m}^{\text{MLE}}$ estimated by the MLE algorithm after the last TCP iteration) with respect to the actual fluorophore position $\left({\bar{r}}_{m}\right)$ for the different settings. We also considered localization algorithms based on multiple pattern repeats within each TCP iteration (Fig. 3B), wherein fluorescence photon counts from each beam position, $\bar{n}$ , are accumulated over the repeats. Here, we used the same MLE localization procedure and excitation powers as in Fig. 3A, but with *t*_dwell_ distributed over five pattern repeats (*t*_dwell_ divided by 5 in each repeat). Overall, the simulation outcomes (Fig. 3, A and B) suggest that fluorophore blinking can substantially affect the localization precision in MINFLUX experiments. For the shorter *t*_dwell_ (5 μs or 30 μs) and lower powers (1 to 10 μW, corresponding to peak irradiances of 0.7 to 6.8 kW/cm^2^) (red-marked squares in Fig. 3, A and B), the total possible excitation time of a completed localization procedure, $t_{\text{total}}$ , will typically be smaller than the time needed for ${\dot{R}}^{-}$ buildup in the fluorophore (four TCP iterations, each with seven beam positions yield $t_{\text{total}}=4\times 7\times t_{\text{dwell}}$ ). The major part of the spread in the ${\bar{r}}_{m}^{\text{MLE}}$ distributions is thus rather caused by photoisomerization, which takes place on a time scale well within $t_{\text{total}}$ , and where the spread is diminished with the number of TCP iterations (fig. S9). Similarly, comparing localization procedures with short $t_{\text{dwell}}$ and low powers between Fig. 3 (A and B, red boxes), noticeable improvement in precision can be seen when N and P populations are averaged over several (here five) pattern repeats. This is consistent with observations that by spreading the beam dwell time, *t*_dwell_, at each beam position over multiple repeats one may obtain an averaging effect of the blinking kinetics (*17*). Next, for localization procedures with longer $t_{\text{dwell}}$ (150 or 500 μs), and especially when combined with higher excitation powers (10 to 30 μW), an additional crescent-shaped localization error pattern emerges, displaying an appreciable loss of accuracy in the localizations (Fig. 3, A and B). Within these longer $t_{\text{dwell}}$ , multiple transitions between N and P can take place and effects on $\bar{n}$ from photoisomerization will be largely averaged out. However, the much slower transitions to and from ${\dot{R}}^{-}$ will not average out to the same extent. Yet, relaxation into ${\dot{R}}^{-}$ can be expected to take place well before the localization procedure is completed ( $t_{\text{total}}$ ). This will skew $\bar{n}$ and move the TCP center to a next estimated position ( ${\bar{r}}_{m}^{\text{MLE}}$ ) far away from the actual fluorophore position ( ${\bar{r}}_{m}$ ). Once generated, the long-lived ${\dot{R}}^{-}$ state may often not relax back within $t_{\text{total}}$ . Then, $\bar{n}=0$ and subsequent iterations cannot correct ${\bar{r}}_{m}^{\text{MLE}}$ (fig. S10). Implementing the pattern repeat leads to an overall improvement in the localization, with less inaccurate localizations, and (except for the highest excitation starting power and $t_{\text{dwell}}$ applied) with most localizations centered around the actual fluorophore position (Fig. 3, B compared A). This improvement is likely not a result of an overall lowered ${\dot{R}}^{-}$ population generated but rather suggests that with pattern-repeats fluorescence is detected over a larger number of beam positions before relaxation into the ${\dot{R}}^{-}$ state takes place. Nonetheless, also with the pattern-repeat applied, a considerable fraction of the localizations remained inaccurate. From the localization error patterns of DL755 (Fig. 3, A and B), using the lowest $t_{\text{dwell}}$ (5 μs) and laser power (1 μW) seems to minimize the localization errors. In practice, however, very few fluorescence photons would then be generated, making it difficult to distinguish these photons above typical background levels.

For reference, we also performed the same set of simulations for AF647 (fig. S11), for the same conditions, and based on the photophysical transition rates, as determined for AF647 (table S1). For the shorter $t_{\text{dwell}}$ and lower excitation powers AF647 displays somewhat larger errors than DL755. This likely reflects the faster and more prominent buildup of photoisomerized P states in AF647. On the other hand, for longer $t_{\text{dwell}}$ and higher excitation powers, a lower fraction of inaccurate localizations was found in the AF647 simulations, likely attributed to the lower extent of ${\dot{R}}^{-}$ state formation in AF647 compared to in DL755 (Fig. 1, E and F). For AF647, we find that the pattern repeat improved the localizations more than for DL755 (fig. S11B), consistent with its more prominent photoisomerization and lower tendency to undergo photoreduction (which would be less remedied by the pattern repeat).

Our simulations show that the spatial distribution of inaccurate localizations around the correct fluorophore location is largely influenced by the excitation history, at what stage in a TCP iteration the fluorophore transits into a dark ( ${\dot{R}}^{-}$) state, and the beam positions preceding that stage. This is clearly illustrated by reversing the TCP direction, which strongly affects in what angle from ${\bar{r}}_{m}$ inaccurate localizations are found (fig. S12). Similarly, the location of the center of the TCP relative to ${\bar{r}}_{m}$ at iteration 1 naturally influences the excitation history and thus also affects how inaccurate localizations distribute around ${\bar{r}}_{m}$ (see simulated examples in fig. S13).

It is worth noting that localization errors attributed to fluorophore blinking, as simulated above, are closely linked to the single fluorophore readout practiced in MINFLUX localization procedures, which places the fluorophore in a photophysical nonequilibrium. If an ensemble of DL755 fluorophores would occupy ${\bar{r}}_{m}$ instead of a single fluorophore, the photophysical states will approach ensemble equilibria as the TCP iterations progress. The fluorescence generated would then show a closer to linear dependence on the excitation rate experienced at ${\bar{r}}_{m}$ , resulting in more accurate and precise localizations, especially with a pattern repeat implemented, in some instances even similar to localizations of nonblinking single fluorophores (figs. S14 to S16). Bringing in ensemble features by multifluorophore labeling of molecular targets or by embedding multiple fluorophores into beads would thus in principle offer a strategy to reduce localization errors in MINFLUX imaging. However, the fluorophores would then need to blink independently and can then in practice not be too close to one another. At the same time, the more separated the fluorophores, the more extended their (common) target location, leading to possible steric effects and compromised localization precision.

### Strategies to overcome blinking-induced localization errors

**Fig. 4.**
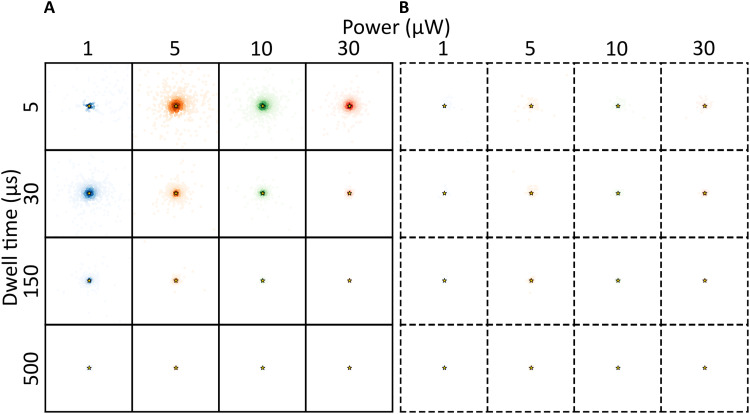
Maps of estimated locations from simulations of a hypothetical DL755 like fluorophore, with no long-lived redox state. The fluorophore is considered at position (1 nm, 1 nm) (golden star) following an iterative MINFLUX localization. The figure shows simulated localization estimates (colored dots) for different powers, different beam dwell times and without (**A**) and with (**B**) pattern repeats (dashed boxes, pattern repeat of 5). With pattern repeats, most of the estimates are very close to the actual position with similar localization errors between different powers and beam dwell times. The ROI (each box) has the dimensions −100 to +100 nm in the *x* and *y* directions. The difference in color transparency shows the density of estimated locations, calculated using a KDE with a Gaussian kernel.

The simulations presented above suggest that fluorophore blinking can substantially contribute to localization errors in MINFLUX imaging experiments. In commercial MINFLUX instruments, automatized, field-programmable gated array (FPGA)-based control and assessment of the photon statistics of a localized fluorophore in between iterations are typically implemented (*34*). Inaccurate localizations as found in our simulations might then prompt the system to abort further TCP iterations and look for new fluorophores available in the field of view. Fluorophore blinking can thus also be a possible reason for the lowered number of final localizations observed in some MINFLUX experiments (*7*, *8*). For the studied cyanine fluorophores, our simulations suggest that transitions to ${\dot{R}}^{-}$ is the major source of blinking-induced localization errors. To further verify this, we simulated 1000 localizations of a hypothetical DL755, for the same conditions as for the simulations above, with isomerization kinetics as determined from the TRAST measurements (Fig. 1G and table S1), but now with no transitions to T or ${\dot{R}}^{-}$ . We then find that the localization precision is better for all conditions tested, and with negligible inaccurate localizations (Fig. 4A). The localization precision improved with longer $t_{\text{dwell}}$ , higher laser power (resulting in shorter isomerization relaxation times), and with a pattern repeat included (Fig. 4B). These improvements are consistent with a larger extent of equilibration/relaxation into the P state for each beam location within a TCP, and that this buildup is averaged over a TCP iteration by several pattern repeats. Corresponding simulations of a hypothetical isomerization-only AF647 showed similar results as for DL755 (fig. S17), further verifying that ${\dot{R}}^{-}$ buildup is a major reason for blinking-induced localization errors of cyanines in MINFLUX experiments.

**Fig. 5.**
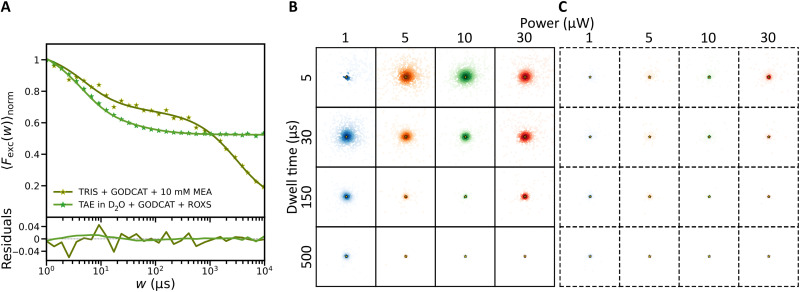
Effect of ROXS buffer on fast blinking and estimated localizations from simulations of DL755. (**A**) TRAST curves recorded for DL755 (conjugated to an antibody) in a STORM switching buffer (tris, GODCAT, and MEA) and for DL755 (conjugated to an imager DNA strand) in a deuterated redox buffer (TAE in D_2_O, GODCAT, and ROXS) (see Materials and Methods for details) (excitation intensity, $I_{\text{exc}}$ = 4.9 kW/cm^2^). (**B** and **C**) Maps of simulated final estimated locations (colored dots) for DL755 in deuterated redox buffer, with minimal long-lived redox state population, at position (1 nm, 1 nm) (golden star) following an iterative MINFLUX localization. The figure shows simulated localization estimates for different powers, different beam dwell times and without (B) and with (C) pattern repeats (dashed boxes, pattern repeat of 5). The ROI (each box) has the dimensions −100 to +100 nm in the *x* and *y* directions. The difference in color transparency shows the density of estimated locations, calculated using a KDE with a Gaussian kernel.

As a strategy to suppress localization errors and ${\dot{R}}^{-}$ buildup in practice, we explored the use of different redox buffers (fig. S18). We found that replacing MEA with ascorbic acid (AA; 1 mM) and methyl viologen (MV; 1 mM) as a reducing and oxidizing system (ROXS) (*35*) into the imaging buffer [deoxygenated TAE (tris, acetic acid, and EDTA) buffer with GODCAT] resulted in an almost complete suppression of ${\dot{R}}^{-}$ buildup in DL755 (Fig. 5A). For AF647, a similar effect was observed from the ROXS, although the ${\dot{R}}^{-}$ buildup to be suppressed was smaller than in the NIR cyanines (fig. S18C). With the ROXS added, DL755 is very similar to the hypothetical isomerization-only DL755 fluorophore considered in the simulations in Fig. 4. Corresponding MINFLUX localization simulations based on fitted transition rates to the TRAST curves from DL755 in the ROXS resulted in similar strongly decreased blinking-attributed localization errors (Fig. 5B).

Another aspect of the added ROXS is that it has also been found to suppress (irreversible) photobleaching (*35*). In our simulations, we did not include photobleaching, since this was not directly observed within the relaxation times covered in the TRAST measurements (up to 10 ms), and since typical emission ON times of single molecules in MINFLUX experiments are much longer (10 ms or longer) than the dwell and iteration times simulated. However, by adding a ROXS buffer one can expect typically less photostable NIR fluorophores to tolerate higher laser irradiances, which can further reduce isomerization-attributed localization errors in MINFLUX experiments (Fig. 5, B and C), generate higher fluorescence photon numbers, $\bar{n}$ , as a basis for the localization, and improve signal-to-background conditions. Another, orthogonal strategy to improve fluorophore brightness, particularly in NIR heptamethine cyanines, is to replace water with heavy (deuterated) water in the samples (*25*, *36*, *37*). Our TRAST experiments showed a two- to threefold increase in the fluorescence signal of DL755 by use of heavy water, a corresponding increase in the fluorescence lifetime, and a concomitant decrease in the isomerization relaxation time (fig. S19). These effects are all in agreement with a lower hydrogen bond–mediated quenching of excited singlet states, of both the *N* and *P* states.

Before ${\dot{R}}^{-}$ state suppression can be used as a strategy to improve localization in MINFLUX imaging, it should be borne in mind that two paths can be taken following ${\dot{R}}^{-}$ formation (*32*). While faster back-electron transfer upon thiol-cyanine radical pair dissociation results in prominent blinking artefacts in the MINFLUX localization, this is not expected for the transitions into long-lived thiol-cyanine adducts. In contrast, the slow (milliseconds to seconds), low duty cycle switching based on this adduct formation is a requirement for the single-molecule localization, in MINFLUX as well as in STORM imaging (*14*). Hence, by suppressing ${\dot{R}}^{-}$ state formation, other means will be needed to perform this slow timescale fluorophore switching. One means is to replace the intrinsic (slow) switching within the fluorophores themselves by the point accumulation for imaging in nanoscale topography (PAINT) method (*38*), more specifically by fluorophore-labeled single-strand DNA (DNA imager strands) transiently binding to complementary strands (DNA docking strands) attached to the target molecules (DNA-PAINT) (*39*). Thereby, several constraints on the fluorophores and their switching kinetics can be relieved in MINFLUX experiments (*40*). While practically eliminating ${\dot{R}}^{-}$ state formation with a balanced redox buffer, we can then specifically replace the slow, low duty cycle switching based on cyanine-thiol adduct formation with an extrinsic switching based on DNA-PAINT.

### Experimental demonstration of NIR-MINFLUX imaging

**Fig. 6.**
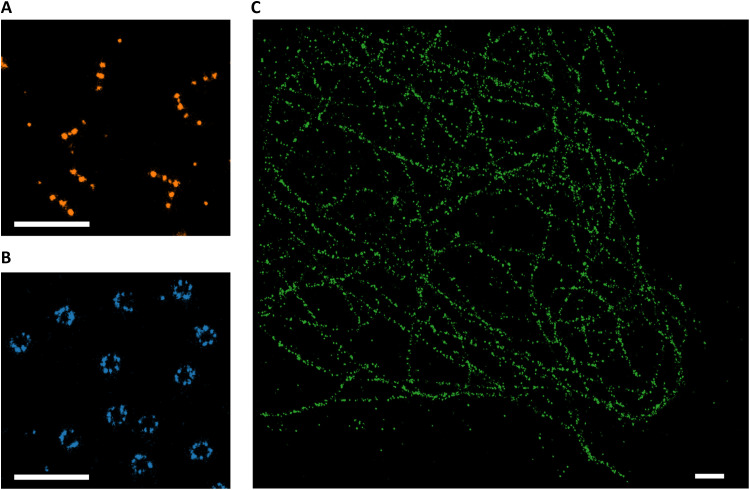
NIR DNA-PAINT MINFLUX images. DNA-PAINT MINFLUX images of (**A**) DNA origami nanorulers with DNA docking strands located at three positions 80 nm apart and with CF750-labeled complementary imager DNA strands, (**B**) Nup96 in U2OS cells labeled with docking strand tagged anti-GFP nanobody and then using AF750-labeled complementary imager DNA strands, and (**C**) tubulin in A549, lung epithelial cells labeled with docking strand tagged secondary nanobody and then using DL755-labeled complementary imager DNA strands. The imaging buffer of all three images contained a deuterated redox buffer (TAE in D_2_O, GODCAT, and ROXS); see Materials and Methods for details. Scale bars, 500 nm

To achieve NIR MINFLUX imaging, we adapted the imaging buffers to suppress ${\dot{R}}^{-}$ buildup in the cyanine fluorophores, as described above, and implemented DNA-PAINT as an external slow switching mechanism. Binding and dissociation of fluorophore-labeled imager strands to the targets can thus replace the intrinsic low duty cycle on-off switching within the fluorophores themselves (mediated via a ${\dot{R}}^{-}$ buildup in long-lived cyanine-thiol adducts), which otherwise is needed for the single-molecule localization. As in antibody conjugation (fig. S4), fluorophore labeling to DNA strands can slightly alter the isomerization and ${\dot{R}}^{-}$ state relaxation rates (less than 20%). However, from simulations, we see that this will not affect the extent of blinking-induced localization errors. Moreover, the typical binding time for the DNA-PAINT imager and docking strands is 100 ms (see Materials and Methods), more than an order of magnitude slower than the back-electron transfer relaxation, and also much slower than the slowest localization procedures simulated (Figs. 3 to 5). Thus, we can rule out artefacts on the localization by the DNA-PAINT switching. For demonstration, we imaged DNA origami nanorulers (GATTA PAINT 80, GATTAquant) immobilized on a cover glass, where imager strands labeled with CF750 (Fig. 6A) or with DL755, AF750, or AF647 (fig. S21) could transiently bind to complementary DNA strands (docking strands) at three equidistant (80 nm) sites on the nanorulers (see Materials and Methods). The imaging was performed with a MINFLUX platform from Abberior Instruments, modified to incorporate NIR excitation (750 nm) and detection (see Materials and Methods). Since ROXS buffer systems have previously been found to reduce photobleaching (*35*), and also to protect DNA docking strands from reactive oxygen species damage (*41*), and since no noticeable ${\dot{R}}^{-}$ or triplet-state buildup was observed (Fig. 5A), higher excitation irradiances could be tolerated in the imaging experiments. Thereby, higher fluorescence photon numbers, $\bar{n}$ , could be obtained as a basis for the localization. For comparison, using instead a commercially available DNA-PAINT imaging buffer, confirmed by TRAST measurements not to suppress ${\dot{R}}^{-}$ buildup in DL755 (fig. S20), resulted in images with almost no successful localizations (neither for AF750, CF750, and AF647; fig. S21). These findings are well in agreement with the simulation results, how the use of a redox-balanced buffer can strongly reduce localization errors (Fig. 5, B and C) and indicate that when a regular DNA-PAINT buffer is used large errors in the localizations are generated, which are discarded by the imaging software (fig. S21). Next to nanoruler images, we also applied the same combination of a balanced redox buffer (ROXS) and DNA-PAINT to demonstrate NIR-MINFLUX imaging in cells. Figure 6 (B and C) shows images of nuclear pore complexes (Nup96) in U2OS cells (with AF750-labeled imager strands) and tubulin in A549 lung epithelial cells (with DL755-labeled imager strands), respectively, both targeted by antibodies with corresponding docking strands (see Materials and Methods for further details). By MINFLUX imaging of Nup96 (with DL755-labeled imager strands) in our redox-balanced buffer (in which not all localizations were discarded), we further investigated effects of excitation power, beam dwell time, and pattern repeats on the localization. As expected, and in agreement with the simulations (Figs. 4 and 5), we found that the localization precision and the number of valid localizations in the images increased with higher excitation powers, longer dwell times, and pattern repeats (fig. S22 and table S2).

Since DNA-PAINT can provide the means for an extrinsic, low duty cycle, slow switching, NIR fluorophores other than heptamethine cyanines can also be considered for MINFLUX imaging, without concern about their intrinsic slow switching properties. As one of a very few noncyanine fluorophores emitting in the NIR (excited in the visible), we investigated the oxazine fluorophore ATTO 700. In contrast to the cyanine fluorophores studied, no substantial photoinduced redox transitions were observed for ATTO 700 in a regular phosphate-buffered saline (PBS) buffer, nor in the same commercially available DNA-PAINT imaging buffer as used above (fig. S23A). Using the latter buffer, with ATTO 700-labeled imager strands and the same nanorulers as above, we could also successfully perform DNA-PAINT MINFLUX imaging (fig. S23B). Using the same ROXS buffer system as above, however, ATTO 700 displayed prominent redox-state relaxations (fig. S23A) in a similar time range as for the cyanines, and then only very sparse localizations were found in the recorded MINFLUX images (fig. S23C). This further illustrates that redox-state transitions within typical time ranges for the TCPs, in cyanines as well as in other fluorophores, represent a major limiting factor in MINFLUX imaging. It further shows the importance of a redox buffer, balanced to the fluorophore(s) used. Moreover, if several fluorophores are to be imaged, they should be compatible with the same redox buffer, i.e., their redox-state transitions should all be possible to suppress with the same redox buffer. Characterizing these transitions by TRAST imaging and subsequent simulations offers a general procedure, whereby sample and excitation conditions can be identified such that localization precisions and probabilities in MINFLUX imaging can be strongly improved. Thereby, additional fluorophore categories, including NIR heptamethine fluorophores (AF750, DL755, and CF750) and oxazines (ATTO 700), can become eligible for use.

While the use of a balanced redox buffer is a prerequisite for NIR MINFLUX imaging (fig. S21), identifying a proper buffer suppressing blinking can also strongly improve the localization performance for other SMLM approaches. This is illustrated in fig. S24, from DNA-PAINT images of nanoruler samples [taken by a DNA-PAINT total internal reflection fluorescence (TIRF) microscope, (*42*) adapted for operation in the NIR using DL755-labeled imager strands] in different redox buffers. In these experiments, use of a well-adapted redox buffer (the deuterated ROXS buffer) resulted in improved localization precision, comparable to that obtained by MINFLUX imaging on the same sample (see fig. S24 for further comparison). As expected, compared to MINFLUX, substantially higher imaging speeds were reached with TIRF-based DNA-PAINT (given its massively parallel localization concept, over a much larger field of view), while MINFLUX needed a substantially lower number of photons for each localization (fig. S24C).

## DISCUSSION

Here, the blinking properties of three heptamethine cyanines, representing the by far dominating category of fluorophores used for NIR imaging were systematically studied over timescales (1 μs to 10 ms), and under labeling and sample conditions relevant for MINFLUX imaging. Similarly, we also studied AF647 as a benchmark fluorophore emitting in the far red. From the acquired data, photodynamic models with rate parameters were determined and next used in simulations of the fluorophore blinking behavior under representative MINFLUX excitation conditions. Our study shows that faster time scale (microseconds to milliseconds) blinking of the fluorophores, especially from photoinduced redox-state transitions, can lead to substantial localization errors and is a major underlying reason for the lowered localization probability experienced in MINFLUX measurements. These problems can be alleviated by use of a balanced redox buffer suppressing such transitions, and by then also replacing the concomitant redox-coupled slow switching of the fluorophores (as needed for the localization) by a DNA-PAINT approach, NIR-MINFLUX imaging can be realized. The overall procedure allowing NIR-MINFLUX imaging to be realized, and the kinetic models for how the use of a balanced redox buffer together with DNA-PAINT affect the fluorophore blinking and switching is summarized in fig. S25. Operation in the NIR can be an advantage for MINFLUX imaging applications in more scattering and autofluorescent media, such as in tissues, where higher background and scattering may be an issue. Our findings are relevant for not only MINFLUX imaging but also MINFLUX tracking applications, as well as for other SRM techniques incorporating time-modulated illumination patterns in the localization (*18*–*20*). They also allow us to define different strategies to minimize localization errors and open for the use of a broader range of fluorophores in MINFLUX imaging and in SRM in general.

## MATERIALS AND METHODS

### Fluorophore sample preparation

Stock solutions of AF647 *N*-hydroxysuccinimide (NHS) ester (Invitrogen, USA), D755 NHS ester (DL755, Thermo Fisher Scientific, USA), AF750 NHS ester (Invitrogen, USA), and CF750 succinimidyl ester (Sigma-Aldrich, USA) were prepared in DMF and stored at −20°C and then diluted in different solvents just before measurements to a final concentration of around 500 nM.

For the TRAST measurements on antibody-conjugated dyes, all dyes were conjugated to AffiniPure sheep anti-mouse IgG (H + L) (Biozol, Germany) with a degree of labeling between 0.1 to 0.2 to be assured that there was just one fluorophore on each antibody.

The imager strand F1 was purchased with dye (AF647 and ATTO 700) conjugation from Massive Photonics. DL755, AF750, and CF750 were conjugated to the oligonucleotide in PBS, through a reaction with fivefold excess of NHS-conjugated dyes over DNA, with 50 mM sodium bicarbonate (NaHCO_3_, pH 9) at room temperature (RT), on a shaker (600 rpm) for 2 hours. After reaction, 0.1× volume of 3 M sodium acetate (pH 5.2) and 2.5× volume of ethanol were added and kept at −20°C for 2 hours, allowing for DNA precipitation. The mixture was then centrifuged at 14,000*g* for 20 min and the supernatant with free dyes was removed. The remaining pellet was then resuspended, washed three times with 70% ethanol, centrifuged at 14,000*g* for 5 min each time, then dried, resuspended, and stored at −20°C in tris EDTA buffer. Typical binding time of these imager strands to the corresponding docking strands stated by the manufacturer is 100 ms.

### Buffer solutions

The buffers used in the TRAST and MINFLUX imaging measurements were as follows (all chemicals purchased from Sigma-Aldrich, USA, unless stated otherwise)

#### STORM switching buffer

Fifty millimolar tris buffer, 10 mM NaCl, GODCAT [10% w/v d-(+)-glucose, catalase (64 μg/ml; C100) and glucose oxidase (0.4 mg/ml; G2133)], and 10 mM mercaptoethylamine (MEA).

#### ROXS redox buffer

TAE (2.5×) in H_2_O or in D_2_O for a deuterated buffer, 2 M NaCl, GODCAT [1% w/v d-(+)-glucose, catalase (50 μg/ml), and glucose oxidase (0.6 mg/ml)], and ROXS (1 mM l-AA and 1 mM methyl viologen dichloride hydrate). Optionally, 0.05% Tween 20 was added to reduce surface stickiness.

#### Commercial DNA-PAINT imaging buffer

Massive Photonics Imaging buffer (1×; Massive Photonics).

#### Trolox redox buffer

TAE (2.5×) in H_2_O, 2 M NaCl, GODCAT [1% w/v d-(+)-glucose, catalase (50 μg/ml), and glucose oxidase (0.6 mg/ml)], 5 mM trolox [200 mM stocks of (±)-6-hydroxy-2,5,7,8-tetramethylchromane-2-carboxylic acid was prepared in dimethyl sulfoxide and kept at −20°C for less than 6 months].

### Nanoruler sample preparation

DNA origami–based nanorulers (GATTA-PAINT 80, GATTAquant GmbH) with three DNA-PAINT F1 docking sites (Massive Photonics) with 80 nm distance between the sites were immobilized in an eight-well glass-bottom chamber (Ibidi μ slide chamber) according to manufacturer instructions. In brief, the chamber was washed with PBS three times (3 min each) before adding bovine serum albumin (BSA)–biotin solution (1 mg/ml) in PBS for 5 min. After incubation with BSA-biotin, the solution is removed, 3× washed with PBS, and incubated with neutravidin solution (1 mg/ml) for 5 min. The chamber is then washed three times with PBS, supplemented with 10 mM magnesium chloride (so-called immobilization buffer, IB). The nanoruler solution is then diluted 1:3 with IB and incubated in the chamber for 20 min. The chamber is again washed three times with IB. Undiluted nanorod dispersion (100 μl; A12-40-980-CTAB-DIH-1-25, Nanopartz Inc.) was then incubated for 20 min for stabilization of samples during MINFLUX imaging. The chamber was then washed three times with IB and replaced with appropriate imaging buffers with imager strands and with 20 mM MgCl_2_ for stabilization of DNA nanorulers. DNA-PAINT MINFLUX was performed with μ slides in air saturated conditions, since the photophysical environment provided by the redox buffer was measured to be preserved for at least 3 hours (fig. S26).

### Cellular sample preparation for Nup96 imaging

The homozygous U2OS-CRISPR-NUP96-mEGFP, clone 195 (*43*) were obtained from CLS/Cytion (CLS Cell Lines Service GmbH). U2OS cells were cultivated in McCoy’s 5a medium (Thermo Fisher Scientific), supplemented with penicillin (100 U ml^−1^), streptomycin (100 μg ml^−1^), 1 mM Na-pyruvate, and 10% (v/v) fetal bovine serum (FBS; Invitrogen) at 37°C, 5% CO_2_. The cells were cultured for 1 day on cover slips (Marienfeld) or in eight-well chambered coverslips (Ibidi μ slide chamber) and fixed in prewarmed 4% paraformaldehyde in PBS for 10 min. Fixed cells were permeabilized with 0.5% (v/v) Triton X-100 in PBS for 5 min. Cells were then blocked in antibody incubation buffer (Massive Photonics) for 60 min. After that, the cells were incubated for 1 hour at RT with the MASSIVE-TAG-X2 single domain nanobody against green fluorescent protein (GFP; clones: 1H1 and 1B2) conjugated with F1 docking strands (Massive Photonics) in antibody incubation buffer (Massive Photonics) at a dilution of 1:100. The cells were then washed three times with 1× washing buffer (Massive Photonics) and incubated with 200-nm gold beads (Sigma-Aldrich) without dilution for 5 min. As the last step, the sample was again washed three times with 1× washing buffer (Massive Photonics).

### Cellular sample preparation for tubulin imaging

A549, lung epithelial cells were cultured in Dulbecco’s modified Eagle’s medium (41965039, Gibco, USA), supplemented with 10% v/v FBS (S1810, Biowest, France) and 1% v/v penicillin-streptomycin solution (SV30010, HyClone, USA), at 37°C, 5% CO_2_. Before measurement, the cells were seeded on eight-well glass-bottom chamber (Ibidi μ slide chamber) and cultured for 24 hours, after which the medium was removed, and cells were washed three times with PBS. Then, cells were fixed and permeabilized with 4% paraformaldehyde, 0.1% glutaraldehyde, and 0.5% Triton X-100 in PBS for 15 min. The cells were washed three times with PBS before blocking with 1% BSA in PBS for 40 min, then incubated with anti–α-tubulin monoclonal antibody (TU-01) produced in mouse (MA-19162, Invitrogen, USA) for 1 hour with a dilution of 1:100 in Massive Photonics antibody incubation buffer. The cells were then washed three times with 1× washing buffer (Massive Photonics) before incubating with FluoTag-XM-QC Anti-Mouse single-domain nanobody with docking site F1 (Massive Photonics) at a dilution of 1:100 in antibody incubation buffer (Massive Photonics) for 1 hour. After another set of washing steps, the samples were incubated with 200-nm gold beads (Sigma-Aldrich) without dilution for 10 min, allowing sample stabilization during MINFLUX measurements. The sample was again washed three times with 1× washing buffer (Massive Photonics).

### TRAST spectroscopy

TRAST measurements were carried out on a home-built TRAST setup based on an inverted epifluorescence microscope. In short, fluorophores were excited by the beam of a diode laser at 638 nm (140 mW, Cobolt, 06-MLD) or at 750 nm (500 mW, MBP communication Inc., Canada), passing an appropriate excitation filter [FF01-637/7 (Semrock) or ET740/40x (Chroma), respectively]. The 638-nm laser beam was modulated directly by external triggering, and the 750-nm laser beam by an acousto-optic modulator (MCQ110-A1,5-IR, AA Opto Electronics, France). The expanded laser beam was defocused by a convex lens, reflected by a dichroic mirror (ZT405/473/559/635/748rpc-UF3 from Chroma), and then focused close to the back aperture of the objective (alpha Plan-Fluar 100×/1.45 Oil, ZEISS, USA) to produce a wide-field illumination in the sample [beam waist ω_0_ = 15 to 25 μm (1/e^2^ radius)]. The fluorescence signal was collected by the same objective, passed through the same dichroic mirror and a filter [638 notch filter or FF02-809/81 (Semrock) emission filter, respectively] to remove scattered laser light, and then fed to a digital scientific complementary metal-oxide semiconductor camera (ORCA-Fusion BT, Hamamatsu Photonics, Japan). The experiments were controlled and synchronized by custom software implemented in Matlab. A digital I/O card (PCI-6602, National Instruments) was used to trigger the camera and generate pulse trains sent to the AOM driver unit.

### MINFLUX imaging

MINFLUX imaging was performed with a platform from Abberior Instruments [see (*34*)], prototyped with a NIR excitation laser (750 nm, 500 mW, MBP Communication Inc., Canada) and with a single-photon avalanche detector (SPAD) with enhanced NIR detection (Excelitas, SPCM-NIR). Data acquisition and instrument control were done using the Imspector software (version 16.3.16297M-minflux, Abberior Instruments) and Imspector sequence Imaging2D (table S3) was used for 2D imaging. A region of interest (ROI) in the samples was selected, displaying sufficient photoswitching upon confocal imaging. Once a ROI was selected, the stabilization system was activated, and the starting excitation power (power for the first iteration) was selected before starting the MINFLUX measurements. For the ATTO 700 and AF647 measurements, laser excitation at 640 nm was used, with a starting laser power of ~210 μW (measured at the back aperture of the objective). On the detection side, a regular SPAD (Excelitas, SPCM-AQRH-13-FC) was used with emission filter (685 to 720 nm for ATTO 700, 650 to 720 nm for AF647) and a pinhole size of 0.83 AU. For the NIR heptamethine cyanine measurements, a 750-nm laser was used, with a starting laser power of ~180 μW, a NIR-SPAD (Excelitas, SPCM-780-14-FC) for detection (emission filter, 770 to 855 nm) and a pinhole size of 1.1 AU. The MINFLUX localizations, obtained from the Imspector software were drift corrected using COMET (cost-function optimized maximal overlap drift estimation) (*44*) and rendered in Python with a bin size of 5 nm and with a color map of 0 to 5. The median localization precision and Fourier ring correlation end point resolution (table S4) were obtained by analyzing the MINFLUX localization data using an open source analysis software pyMINFLUX (*45*).

### Fluorescence lifetime measurements

Fluorescence lifetime measurements were performed by time-correlated single-photon counting (TCSPC); see text S2 for details.

### Simulations of MINFLUX localizations of individual fluorophores

Simulations are described in the Results and with further details in text S6 (*46*–*48*).

## References

[1] S. W. Hell, J. Wichmann, Breaking the diffraction resolution limit by stimulated-emission–Stimulated-emission-depletion fluorescence microscopy. Opt. Lett. 19, 780–782 (1994). 19844443 10.1364/ol.19.000780

[2] T. A. Klar, S. Jakobs, M. Dyba, A. Egner, S. W. Hell, Fluorescence microscopy with diffraction resolution barrier broken by stimulated emission. Proc. Natl. Acad. Sci. U.S.A. 97, 8206–8210 (2000). 10899992 10.1073/pnas.97.15.8206 PMC26924

[3] E. Betzig, G. H. Patterson, R. Sougrat, O. W. Lindwasser, S. Olenych, J. S. Bonifacino, M. W. Davidson, J. Lippincott-Schwartz, H. F. Hess, Imaging intracellular fluorescent proteins at nanometer resolution. Science 313, 1642–1645 (2006). 16902090 10.1126/science.1127344

[4] M. J. Rust, M. Bates, X. W. Zhuang, Sub-diffraction-limit imaging by stochastic optical reconstruction microscopy (STORM). Nat. Methods 3, 793–796 (2006). 16896339 10.1038/nmeth929 PMC2700296

[5] F. Balzarotti, Y. Eilers, K. C. Gwosch, A. H. Gynnå, V. Westphal, F. D. Stefani, J. Elf, S. W. Hell, Nanometer resolution imaging and tracking of fluorescent molecules with minimal photon fluxes. Science 355, 606–612 (2017). 28008086 10.1126/science.aak9913

[6] K. C. Gwosch, J. K. Pape, F. Balzarotti, P. Hoess, J. Ellenberg, J. Ries, S. W. Hell, MINFLUX nanoscopy delivers 3D multicolor nanometer resolution in cells. Nat. Methods 17, 217–224 (2020). 31932776 10.1038/s41592-019-0688-0

[7] S. Liu, P. Hoess, J. Ries, Super-resolution microscopy for structural cell biology. Annu. Rev. Biophys. 51, 301–326 (2022). 35119945 10.1146/annurev-biophys-102521-112912

[8] K. Prakash, A. P. Curd, Assessment of 3D MINFLUX data for quantitative structural biology in cells. Nat. Methods 20, 48–51 (2023). 36522506 10.1038/s41592-022-01694-x

[9] L. Feng, W. J. Chen, X. X. Ma, S. H. Liu, J. Yin, Near-infrared heptamethine cyanines (Cy7): From structure, property to application. Org. Biomol. Chem. 18, 9385–9397 (2020). 33191410 10.1039/d0ob01962c

[10] A. P. Gorka, R. R. Nani, M. J. Schnermann, Harnessing cyanine reactivity for optical imaging and drug delivery. Acc. Chem. Res. 51, 3226–3235 (2018). 30418020 10.1021/acs.accounts.8b00384

[11] G. S. Hong, A. L. Antaris, H. J. Dai, Near-infrared fluorophores for biomedical imaging. Nat. Biomed. Eng. 1, 0010 (2017).

[12] H. Blom, J. Widengren, Stimulated emission depletion microscopy. Chem. Rev. 117, 7377–7427 (2017). 28262022 10.1021/acs.chemrev.6b00653

[13] T. Ha, P. Tinnefeld, in *Annual Review of Physical Chemistry*, M. A. Johnson, T. J. Martinez, Eds. (2012), vol. 63, pp. 595–617. 10.1146/annurev-physchem-032210-103340 PMC3736144 22404588

[14] M. Heilemann, S. van de Linde, M. Schuttpelz, R. Kasper, B. Seefeldt, A. Mukherjee, P. Tinnefeld, M. Sauer, Subdiffraction-resolution fluorescence imaging with conventional fluorescent probes. Angew. Chem. Intl. Ed. 47, 6172–6176 (2008). 10.1002/anie.200802376 18646237

[15] G. T. Dempsey, J. C. Vaughan, K. H. Chen, M. Bates, X. W. Zhuang, Evaluation of fluorophores for optimal performance in localization-based super-resolution imaging. Nat. Methods 8, 1027–1036 (2011). 22056676 10.1038/nmeth.1768 PMC3272503

[16] K. C. Gwosch, F. Balzarotti, J. K. Pape, P. Hoess, J. Ellenberg, J. Ries, U. Matti, R. Schmidt, S. J. Sahl, S. W. Hell, Reply to: Assessment of 3D MINFLUX data for quantitative structural biology in cells. Nat. Methods 20, 52–54 (2023). 36522499 10.1038/s41592-022-01695-w

[17] Y. Eilers, H. Ta, K. C. Gwosch, F. Balzarotti, S. W. Hell, MINFLUX monitors rapid molecular jumps with superior spatiotemporal resolution. Proc. Natl. Acad. Sci. U.S.A. 115, 6117–6122 (2018). 29844182 10.1073/pnas.1801672115 PMC6004438

[18] L. S. Gu, Y. Y. Li, S. W. Zhang, Y. H. Xue, W. X. Li, D. Li, T. Xu, W. Ji, Molecular resolution imaging by repetitive optical selective exposure. Nat. Methods 16, 1114–1118 (2019). 31501551 10.1038/s41592-019-0544-2

[19] L. S. Gu, Y. Y. Li, S. W. Zhang, M. G. Zhou, Y. H. Xue, W. X. Li, T. Xu, W. Ji, Molecular-scale axial localization by repetitive optical selective exposure. Nat. Methods 18, 369–373 (2021). 33795876 10.1038/s41592-021-01099-2

[20] P. Jouchet, C. Cabriel, N. Bourg, M. Bardou, C. Poüs, E. Fort, S. Lévêque-Fort, Nanometric axial localization of single fluorescent molecules with modulated excitation. Nat. Photon. 15, 297–304 (2021).

[21] J. Widengren, Fluorescence-based transient state monitoring for biomolecular spectroscopy and imaging. J. R. Soc. Interface 7, 1135–1144 (2010). 20375039 10.1098/rsif.2010.0146 PMC2894879

[22] J. Widengren, in *Fluorescence Microscopy and Spectroscopy in Biology*, M. Amaro, R. Sachl, Eds. (Springer, 2022).

[23] J. Widengren, P. Schwille, Characterization of photoinduced isomerization and back-isomerization of the cyanine dye Cy5 by fluorescence correlation spectroscopy. J. Phys. Chem. A 104, 6416–6428 (2000).

[24] J. Widengren, C. A. M. Seidel, Manipulation and characterization of photo-induced transient states of Merocyanine 540 by fluorescence correlation spectroscopy. Phys. Chem. Chem. Phys. 2, 3435–3441 (2000).

[25] E. Sandberg, J. Piguet, U. Kostiv, G. Baryshnikov, H. Liu, J. Widengren, Photoisomerization of heptamethine cyanine dyes results in red-emissive species: Implications for near-IR, single-molecule, and super-resolution fluorescence spectroscopy and imaging. J. Phys. Chem. B 127, 3208–3222 (2023). 37011608 10.1021/acs.jpcb.2c08016 PMC10108366

[26] E. Sandberg, J. Piguet, H. C. Liu, J. Widengren, Combined fluorescence fluctuation and spectrofluorometric measurements reveal a red-shifted, near-IR emissive photo-isomerized form of cyanine 5. Int. J. Mol. Sci. 24, 1990 (2023). 36768309 10.3390/ijms24031990 PMC9916991

[27] A. K. Chibisov, Triplet states of cyanine dyes and reactions of electron-transfer with their participation. J. Photochem. 6, 199–214 (1976).

[28] M. Levitus, S. Ranjit, Cyanine dyes in biophysical research: The photophysics of polymethine fluorescent dyes in biomolecular environments. Q. Rev. Biophys. 44, 123–151 (2011). 21108866 10.1017/S0033583510000247

[29] Z. Du, J. Piguet, G. Baryshnikov, J. Tornmalm, B. Demirbay, H. Ågren, J. Widengren, Imaging fluorescence blinking of a mitochondrial localization probe: Cellular localization probes turned into multifunctional sensors. J. Phys. Chem. B 126, 3048–3058 (2022). 35417173 10.1021/acs.jpcb.2c01271 PMC9059120

[30] J. Tornmalm, E. Sandberg, M. Rabasovic, J. Widengren, Local redox conditions in cells imaged via non-fluorescent transient states of NAD(P)H. Sci. Rep. 9, 15070 (2019). 31636326 10.1038/s41598-019-51526-w PMC6803634

[31] J. Tornmalm, J. Widengren, Label-free monitoring of ambient oxygenation and redox conditions using the photodynamics of flavin compounds and transient state (TRAST) spectroscopy. Methods 140-141, 178–187 (2018). 29179988 10.1016/j.ymeth.2017.11.013

[32] Y. Gidi, L. Payne, V. Glembockyte, M. S. Michie, M. J. Schnermann, G. Cosa, Unifying mechanism for thiol-induced photoswitching and photostability of cyanine dyes. J. Am. Chem. Soc. 142, 12681–12689 (2020). 32594743 10.1021/jacs.0c03786 PMC8500274

[33] G. T. Dempsey, M. Bates, W. E. Kowtoniuk, D. R. Liu, R. Y. Tsien, X. W. Zhuang, Photoswitching mechanism of cyanine dyes. J. Am. Chem. Soc. 131, 18192–18193 (2009). 19961226 10.1021/ja904588g PMC2797371

[34] R. Schmidt, T. Weihs, C. A. Wurm, I. Jansen, J. Rehman, S. J. Sahl, S. W. Hell, MINFLUX nanometer-scale 3D imaging and microsecond-range tracking on a common fluorescence microscope. Nat. Comm. 12, 1478 (2021). 10.1038/s41467-021-21652-z PMC7935904 33674570

[35] J. Vogelsang, R. Kasper, C. Steinhauer, B. Person, M. Heilemann, M. Sauer, P. Tinnefeld, A reducing and oxidizing system minimizes photobleaching and blinking of fluorescent dyes. Angew. Chem. Intl. Ed. 47, 5465–5469 (2008). 10.1002/anie.200801518 18601270

[36] K. Klehs, C. Spahn, U. Endesfelder, S. F. Lee, A. Fürstenberg, M. Heilemann, Increasing the brightness of cyanine fluorophores for single-molecule and superresolution imaging. ChemPhysChem 15, 637–641 (2014). 24376142 10.1002/cphc.201300874

[37] S. S. Matikonda, G. Hammersley, N. Kumari, L. Grabenhorst, V. Glembockyte, P. Tinnefeld, J. Ivanic, M. Levitus, M. J. Schnermann, Impact of cyanine conformational restraint in the near-infrared range. J. Org. Chem. 85, 5907–5915 (2020). 32275153 10.1021/acs.joc.0c00236 PMC8459201

[38] A. Sharonov, R. M. Hochstrasser, Wide-field subdiffraction imaging by accumulated binding of diffusing probes. Proc. Natl. Acad. Sci. U.S.A. 103, 18911–18916 (2006). 17142314 10.1073/pnas.0609643104 PMC1748151

[39] R. Jungmann, M. S. Avendaño, J. B. Woehrstein, M. J. Dai, W. M. Shih, P. Yin, Multiplexed 3D cellular super-resolution imaging with DNA-PAINT and Exchange-PAINT. Nat. Methods 11, 313–318 (2014). 24487583 10.1038/nmeth.2835 PMC4153392

[40] L. M. Ostersehlt, D. C. Jans, A. Wittek, J. Keller-Findeisen, K. Inamdar, S. J. Sahl, S. W. Hell, S. Jakobs, DNA-PAINT MINFLUX nanoscopy. Nat. Methods 19, 1072–1075 (2022). 36050490 10.1038/s41592-022-01577-1 PMC9467913

[41] M. Scheckenbach, T. Schubert, C. Forthmann, V. Glembockyte, P. Tinnefeld, Self-regeneration and self-healing in DNA origami nanostructures. Angew. Chem. Intl. Ed. 60, 4931–4938 (2021). 10.1002/anie.202012986 PMC7986372 33230933

[42] J. I. Gallea, O. Nevskyi, Z. Kazmierczak, I. Gligonov, T. Chen, P. Miernikiewicz, A. M. Chizhik, L. Reinkensmeier, K. Dabrowska, M. Bates, J. Enderlein, Super-resolution goes viral: T4 virus particles as versatile 3D-bio-nanorulers. Adv. Mater. 37, e2403365 (2025). 39821930 10.1002/adma.202403365 PMC11937993

[43] J. V. Thevathasan, M. Kahnwald, K. Cieslinski, P. Hoess, S. K. Peneti, M. Reitberger, D. Heid, K. C. Kasuba, S. J. Hoerner, Y. M. Li, Y. L. Wu, M. Mund, U. Matti, P. M. Pereira, R. Henriques, B. Nijmeijer, M. Kueblbeck, V. J. Sabinina, J. Ellenberg, J. Ries, Nuclear pores as versatile reference standards for quantitative superresolution microscopy. Nat. Methods 16, 1045–1053 (2019). 31562488 10.1038/s41592-019-0574-9 PMC6768092

[44] e. A. Reinkensmeier, (2024); https://www.smlm.tools/.

[45] A. Ponti, J. C. Arias, T. Horn. (2025). pyMINFLUX (0.6.0). Zenodo. 10.5281/zenodo.15388483.

[46] T. Weihs, *Localization and tracking of single molecules with a MINFLUX microscope for various applications* (Georg August University, Göttingen, 2021).

[47] L. Patel, N. Gustafsson, Y. Lin, R. Ober, R. Henriques, E. Cohen, A hidden Markov model approach to characterizing the photo-switching behavior of fluorophores. Annal. Appl. Stat. 13, 1397–1429 (2019). 10.1214/19-AOAS1240 PMC6957128 31933716

[48] T. Staudt, T. Aspelmeier, O. Laitenberger, C. Geisler, A. Egner, A. Munk, Statistical molecule counting in super-resolution fluorescence microscopy: Towards quantitative nanoscopy. Stat. Sci. 35, 92–111 (2020).

