# Supplementary material for: Near-infrared MINFLUX imaging enabled by suppression of fluorophore blinking
Source: Sci Adv. 2025 Dec 5;11(49):eadw3149. doi: 10.1126/sciadv.adw3149 (PMC12680042; doi:10.1126/sciadv.adw3149)
Supplement: Supplementary file 1 — Supplementary Text S1 to S6 Figs. S1 to S26 Tables S1 to S5 [file sciadv.adw3149_sm.pdf]

Science Advances
AAAS

Supplementary Materials for

Near-infrared MINFLUX imaging enabled by suppression of fluorophore blinking

Chinmaya V. Srambickal *et al.*

Corresponding author: Jerker Widengren, jwideng@kth.se

Sci. Adv. **11**, eadw3149 (2025)
DOI: 10.1126/sciadv.adw3149

This PDF file includes:

Supplementary Text S1 to S6
Figs. S1 to S26
Tables S1 to S5

Supplementary Text

S1. TRAST concept

In TRAST spectroscopy/imaging, the population dynamics of photoinduced, non- or weakly fluorescent, long-lived transient states of fluorescent molecules, such as triplet, photo-isomerized and photo-redox states, are determined from the average fluorescence intensity detected in the sample, when subject to different excitation pulse trains (*21,22*).

Considering a homogeneous fluorophore sample, with concentration, *c*, subject to a rectangular excitation pulse with a constant excitation photon flux, Φ_{exc}, starting at $t = 0$, the recorded fluorescence intensity can be described by

$$F(t) = c \cdot {}^{1}q_F \cdot {}^{1}q_D \cdot \sigma_1 \iiint \left(CEF(\bar{r}) \cdot \Phi_{\mathrm{exc}}(\bar{r}) \cdot \sum_{j=1}^{n} \left(Q_j [A_j](\bar{r}, t) \right) \right) \mathrm{d}V \qquad \text{(S1)}$$

Here, $[A_j]$ denotes the population probability of the i:th photophysical state, ${}^{1}q_F$ and ${}^{1}q_D$ denote the fluorescence quantum yield of state A_1 and the overall detection quantum yield of its emission, respectively. σ_1 is its excitation cross section and $CEF(\bar{r})$ is the collection efficiency function of the setup. With A_1 representing the brightest photophysical state, Q_j then denotes the relative brightness of the other states (*j*=2,…n), compared to that of A_1, and with Q_1 (the brightness of A_1) normalized to one.

At onset of excitation, equilibration between any ground and excited singlet states typically occur with a relaxation time in the nanosecond time range. This so-called anti-bunching relaxation time is typically much faster than the characteristic relaxation(s) of the more long-lived dark transient states. The anti-bunching can thus be disregarded on a µs to ms time scale after onset of excitation, at which relaxations into dark or weakly emissive transient state often take place. Similar relaxations can also be observed in the time-averaged fluorescence signal resulting from a rectangular excitation pulse of duration w

$$\langle F_{\mathrm{exc}}(w) \rangle = \frac{1}{w} \int_0^w F(t)\, \mathrm{d}t \qquad \text{(S2)}$$

when w is increased from the µs to the ms time range. Analyzing how $\langle F_{\mathrm{exc}}(w) \rangle$ varies with w then allows the population kinetics of long-lived photo-induced states of the fluorophore to be determined, which is the general basis for TRAST monitoring.

To obtain sufficient photon counts, even for short w, the fluorescence intensity resulting from an excitation pulse train of *M* identical pulse repetitions is typically recorded. *M* is adjusted to maintain a constant laser illumination time, $t_{ill} = M \cdot w$, for all w. A so-called TRAST curve

is then produced by calculating the time-averaged fluorescence signal during excitation for each pulse train, normalized for a given pulse duration, w_0

$$\langle F_{\text{exc}}(w)\rangle_{\text{norm}} = \left(\frac{1}{M}\sum_{i=1}^{M}\langle F_{\text{exc}}(w)\rangle_i\right)\Big/\left(\frac{1}{M_0}\sum_{i=1}^{M_0}\langle F_{\text{exc}}(w_0)\rangle_i\right) \qquad \text{(S3)}$$

The pulse duration used for normalization, w_0, is chosen to be short enough (typically sub-µs) not to lead to any noticeable build-up of dark transient states, yet longer than the nanosecond anti-bunching rise time of $F(t)$ upon onset of excitation. M_0 refers to the number of pulses used in the normalization.

In the above expression, $\langle F_{\text{exc}}(w)\rangle_i$ represents the total signal collected from the *i*:th pulse in a pulse train, as defined in Eq. S2. By using a low excitation duty cycle, in this work $\eta = 0.001 - 0.01$, fluorophores are allowed to fully recover back to the all-*trans* ground singlet state, S_0, before the onset of the next pulse. In the normalization step of Eq. S3, several parameters used to calculate $F(t)$ in Eq. S1 cancel out. The final expression for $\langle F_{\text{exc}}(w)\rangle_{\text{norm}}$ therefore becomes independent of c as well as of the absolute q_D and q_F values for the emissive species:

$$\langle F_{\text{exc}}(w)\rangle_{\text{norm}} = \frac{\int_{t=0}^{w}\left(\iiint\left(CEF(\bar{r})\cdot\Phi_{\text{exc}}(\bar{r})\cdot\sum_{i=1}^{n}\left(Q_i[A_i](\bar{r},t)\right)\right)\mathrm{d}V\right)dt}{w\iiint\left(CEF(\bar{r})\cdot\Phi_{\text{exc}}(\bar{r})\cdot\sum_{i=1}^{n}\left(Q_i[A_i](\bar{r},0)\right)\right)\mathrm{d}V} \qquad \text{(S4)}$$

S2. Fluorescence lifetime measurements

Fluorescence lifetime measurements were performed by time-correlated single photon counting (TCSPC) using an epi-illuminated, confocal laser scanning microscope (Olympus FV1200). Fluorophore solution samples were excited by the focused beam of a 640 nm diode laser (LDH-D-C-640 , PicoQuant GmbH, Berlin) and of a 750 nm RF tunable laser (SuperK SELECT from NKT-photonics) operated in pulsed mode. The emitted fluorescence was collected back through the microscope objective (UPlanSApo 60x/1.2W, Olympus), passed through a dichroic mirror (ZT405/488/635rpc-UF2, Chroma or T770lpxr-UF2, Chroma), an emission filter (HQ720/150, Chroma, or 809/81 Brightline, Semrock, Semrock), and focused onto a pinhole (50µm diameter) in the back focal plane. The fluorescence signal was finally split and directed on two avalanche photodiodes (Tau-SPAD, PicoQuant GmbH, Berlin). Instrument response functions (IRFs) were determined from the back-reflected light from the laser excitation pulses. The signals were fed into a data acquisition card (Hydraharp 400, Picoquant GmbH), deconvoluted and then fit to an exponential decay based on non-linear least squares minimization (Symphotime, Picoquant GmbH).

S3. Photophysical model and rate equations for the cyanine fluorophores

With the photophysical model shown in Figure 1D (main text), the population probabilities of the different states of the cyanine fluorophores, subject to a constant excitation photon flux of Φ_{exc} starting at time t=0, is given by

$$\frac{d}{dt}\bar{A}(t) = M \cdot \bar{A}(t) \qquad \text{(S5)}$$

Here, $\bar{A}(t) = \left[\, [N](t), [P](t), [T](t), [\dot{R}^-](t)\right]^T$ represents the population probabilities of the all-*trans*, the photo-isomerized, triplet and photo-reduced states of the fluorophore and

$$M = \begin{bmatrix} -(k_{iso}' + k_{isc}') & k_{biso}' & k_T & k_{ox} \\ k_{iso}' & -k_{biso}' & 0 & 0 \\ k_{isc}' & 0 & -(k_T + k_{red}) & 0 \\ 0 & 0 & k_{red} & -k_{ox} \end{bmatrix} \qquad \text{(S6)}$$

is the rate matrix describing the transitions between the states. In the model, it can be assumed that equilibration between the ground and excited singlet states of N and P thus take place on a much faster time scale than the relaxation of $\bar{A}(t)$, i.e. the time scale of the fluorophore dark state transitions and the TRAST experiments (1µs-10ms), and at which also the MINFLUX beam localization procedure typically operates. [*N*] and [*P*] thus denote the total probabilities of the fluorophore to be in either its ground or excited singlet state for N and P, respectively. In the matrix, we can then also assign effective isomerization (k_{iso}'), back-isomerization (k_{biso}') and intersystem crossing (k_{isc}') rates. The effective isomerization rate, from N to P is given by:

$$k_{iso}' = k_{iso} \cdot \frac{\sigma_N \cdot \Phi_{exc}}{\sigma_N \cdot \Phi_{exc} + k_{10}^N} \qquad \text{(S7)}$$

Here, σ_N denotes the excitation cross sections of the singlet ground state of N. k_{10}^N signifies the decay rate from the excited singlet state to the ground singlet state in N, given as the inverse fluorescence lifetime of N, as determined from TCSPS measurements (Section S2). Since k_{10}^P and σ_P could not be individually determined, we defined the back-isomerization rate from P to N by:

$$k_{biso}' = k_{biso} \cdot \frac{\sigma_P \cdot \Phi_{exc}}{\sigma_P \cdot \Phi_{exc} + k_{10}^P} + k_{biso}^{Th} = \left\{k_{10}^P \gg \sigma_P \cdot \Phi_{exc}\right\} = \sigma_{biso} \cdot \Phi_{exc} + k_{biso}^{Th} \qquad \text{(S8A)}$$

, where k_{biso}^{Th} denotes the thermal back-isomerization rate, and where the back-isomerization cross section is defined as:

$$\sigma_{biso} = k_{biso} \cdot \frac{\sigma_P}{k_{10}^P} \qquad \text{(S8B)}$$

Analogous to Eq. S3, the effective intersystem crossing rate from N to T can be defined as:

$$k_{isc}' = k_{isc} \cdot \frac{\sigma_N \cdot \Phi_{exc}}{\sigma_N \cdot \Phi_{exc} + k_{10}^N} \qquad \text{(S9)}$$

The initial condition for Eq. (S1) is

$$\bar{A}(0) = [1\ 0\ 0\ 0\ ]^T \qquad \text{(S10)}$$

, based on the finding that, in absence of excitation, polymethine cyanine fluorophores typically exist in their all-*trans* state (*28*). All fluorophores can thus be assumed to be in the all-*trans* singlet (ground) state before onset of excitation at $t = 0$.

For a rectangular excitation pulse, Φ_{exc} is constant throughout the excitation duration and the matrix M is not time dependent. The general solution to Eq S1 is then

$$\bar{A}(t) = e^{Mt} \cdot \bar{A}(0) \qquad \text{(S11)}$$

The dependence of the detected fluorescence at time, *t*, after onset of excitation is then given by

$$F(t) = {}^{1}q_F \cdot {}^{1}q_D \cdot k_{10}^N \cdot \frac{\sigma_N \cdot \Phi_{exc}}{\sigma_N \cdot \Phi_{exc} + k_{10}^N} \cdot [N](t) + {}^{2}q_F \cdot {}^{2}q_D \cdot k_{10}^P \cdot \frac{\sigma_P \cdot \Phi_{exc}}{\sigma_P \cdot \Phi_{exc} + k_{10}^P} \cdot [P](t)$$

(S12)

, with σ_N and σ_P denoting the excitation cross sections of the N and P state, respectively. ${}^{X}q_F$ is the fluorescence quantum yield and ${}^{X}q_D$ the overall detection quantum yield of the emission from N (X=1) and P (X=2) state, respectively. For the excitation conditions in our study, $k_{10} \gg \sigma_N \cdot \Phi_{exc}, \sigma_P \cdot \Phi_{exc}$, so that we can assume

$$F(t) = {}^{1}q_F \cdot {}^{1}q_D \cdot \sigma_N \cdot \Phi_{exc} \cdot \big([N](t) + Q \cdot [P](t)\big) \quad \text{(S13)}$$

, with $Q = ({}^{2}q_F \cdot {}^{2}q_D \cdot \sigma_{\mathrm{N}})/({}^{1}q_F \cdot {}^{1}q_D \cdot \sigma_{P_2})$ representing the relative brightness of P, compared to N. Since isomerization can take place over several bonds in the polymethine chains of the fluorophores, different photo-isomerized states can likely be populated (*25*). However, compared to isomerization to and from an emissive all-*trans* state, N, transitions between different (non- or weakly emitting) photo-isomerized states yield minor blinking effects. A single state representing all photo-isomerized states, P, is thus sufficient to describe the blinking kinetics, as observed in the TRAST experiments, and as a basis for the simulations of the MINFLUX localization.

S4. TRAST data analysis

Calculation of TRAST curves from the photophysical model

With the transient state population kinetics of the cyanine fluorophores described by the four-state photophysical model shown in Figure 1D, $\bar{A}(t) = \left[\, [N](t), [P](t), [T](t), [\dot{R}^{-}](t)\right]^{T}$ can be assigned to represent the population probabilities of the all-*trans*, the photo-isomerized, triplet and photo-reduced states of the fluorophore, respectively. For the studied cyanine fluorophores, N and the P represent emissive states, where $[N]$ and $[P]$ represent the total population probability of N and P, to be in either their ground or excited singlet states. For a fluorophore subject to a rectangular excitation pulse with constant Φ_{exc} starting at $t = 0$, the fluorescence intensity response then reflects the time dependence of $[N]$ and $[P]$:

$$F(t) = c \cdot {}^{1}q_F \cdot {}^{1}q_D \cdot \sigma_N \iiint \left(CEF(\bar{r}) \cdot \Phi_{\text{exc}}(\bar{r}) \cdot \left([N](\bar{r},t) + Q \cdot [P](\bar{r},t) \right) \right) \mathrm{d}V \quad \text{(S14)}$$

Here, Q is the relative brightness of P compared to N. ${}^{1}q_F$ and ${}^{1}q_D$ denote the fluorescence quantum yield and the overall detection quantum yield of the emission from the excited singlet state of N. σ_N denotes the excitation cross section of the ground singlet state of N.

With all fluorophores in their all-*trans* (N) state at onset of excitation ($\bar{A}(0) = [1\ 0\ 0\ 0\ ]^{T}$, see Eq. S10), the averaged and normalized fluorescence intensities in the recorded TRAST curves can then be written:

$$\langle F_{\text{exc}}(w) \rangle_{\text{norm}} = \frac{\int_{t=0}^{w} \left(\iiint \left(CEF(\bar{r}) \cdot \Phi_{\text{exc}}(\bar{r}) \cdot \left([\mathrm{N}](\bar{r},t) + Q \cdot [\mathrm{P}](\bar{r},t) \right) \right) \mathrm{d}V \right) dt}{w \iiint \left(CEF(\bar{r}) \cdot \Phi_{\text{exc}}(\bar{r}) \right) \mathrm{d}V} \quad \text{(S15)}$$

The time dependence of $[N]$ and $[P]$ for a fluorophore subject to a rectangular excitation pulse with constant Φ_{exc} starting at $t = 0$ is described by Eqs. S5-S13 in section S3.

If [N] and [P] can be assumed to be constant within the detection volume at any specific time, *t*, during a rectangular excitation pulse, then Eq. S15 simplifies to:

$$\langle F_{\text{exc}}(w) \rangle_{\text{norm}} = \frac{1}{w} \int_{t=0}^{w} \left([\mathrm{N}](t) + Q \cdot [\mathrm{P}](t) \right) dt \quad \text{(S16)}$$

Analysis of experimental TRAST data

The data analysis was performed similarly to in previous work,(*25-26,29-31*) adapted to the experimental conditions, samples and models used here (see also Eqs. S5-S16).

A complete TRAST experiment consisted of a stack of 30 fluorescence images. Each image represents the total fluorescence signal from an entire excitation pulse train, captured using a camera exposure time of $t_{\text{exp}} = t_{ill}/\eta$. Images were recorded applying different pulse durations, w, distributed logarithmically between 1 µs and 10 ms. They were measured in a randomized order to avoid bias due to time effects. An additional 10 reference frames, all using

1 μs pulse duration to avoid dark state build-up, were inserted at regular intervals between the 30 main images to track any permanent fluorescence photobleaching of the sample.

The TRAST data were analyzed using a software implemented in Matlab, as previously described (*25-26,29-31*). The recorded TRAST data was first pre-processed by subtraction of the static ambient background and corrected for photobleaching, as described above.

Averaged fluorescence signals, as used in the generation of the TRAST curves (Eq. S3), were calculated within a region of interest (ROI) corresponding to a ~2 μm radius in the focusing plane on the sample, centered on the excitation beam. Since the excitation beam and the excitation photon flux, $\Phi_{\text{exc}}(\bar{r})$, is not fully uniform, a spatial dependence can be expected on the excitation rates and the resulting electronic state populations. The total fluorescence signal on each pixel of the camera then becomes a convolution of $[\text{N}](\bar{r},t) + Q \cdot [\text{P}](\bar{r},t)$ and the microscope collection efficiency function, $CEF(\bar{r})$. By simulating the whole 3D sample volume, and computing the projected 2D image on the camera, it has been found that pre-computing an average observed excitation rate, $\hat{k}_{01}$, for each ROI to be analyzed, speeds up the fitting substantially, without appreciable loss of accuracy (*29-31*). An approximate $\hat{k}_{01}$ could thus be computed once, before fitting starts, by weighting $k_{01}(\bar{r})$ by brightness and collection efficiency, $CEF(\bar{r})$:

$$\hat{k}_{01} = \frac{\iiint k_{01}(\bar{r})\cdot\hat{S}_1(\bar{r})\cdot CEF(\bar{r})\mathrm{d}V}{\iiint \hat{S}_1(\bar{r})\cdot CEF(\bar{r})\mathrm{d}V} \tag{S17}$$

Here, $\hat{S}_1(\bar{r}) = k_{01}(\bar{r})/(k_{10} + k_{01}(\bar{r}))$ represents the population of excited singlet state fluorophores when in an all-*trans* form, N, at onset of excitation, after equilibration between the ground and excited singlet states of N, but before build-up of the other states.

Fitting of photophysical rate parameters was then performed by simulating theoretical TRAST curves using Eqs. S1−S9 and comparing them to the experimental data. The set of rate parameter values best reproducing the experimental data was then found using nonlinear least-squares optimization. In the fit, the excited-state lifetime, τ_{f}, of N, was fixed to its fitted value determined by the TCSPC measurements and with $1/\tau_{\text{f}}$ comprising all deactivation rates from the excited state of N.

S5. Effects of relative brightness of photo-isomerized states on DL755

In recorded TRAST curves from DL755, we found that the amplitude of the fast, photo-isomerization relaxation was lowered the more red-shifted emission band-pass filters were used, see Figure S3 below. We attribute this amplitude effect to a weak, red-shifted emission

from photo-isomerized states of the fluorophores, as recently identified for other cyanine fluorophores (*25,26*). It can be accounted for in the photophysical model by assigning P a relative brightness factor, Q (Eq. S13).

With the emission of the P state shifted towards longer wavelengths compared to the emission from N, the Q factor will increase and the amplitude of the isomerization relaxation in the TRAST curves will decrease. Thus, in principle, an emission filter can also be selected for which Q=1. With such filter selected, no isomerization-related blinking is detected and corresponding localization errors in MINFLUX imaging could be eliminated. However, the overall brightness (from both N and P) would then be lowered. Then, as an alternative, detection over multiple filters in parallel, and then summing 1/Q-factor weighted signal contributions from the different emission filters could also provide a strategy to" neutralize" isomerization-related blinking in the detected signal.

S6. Simulations of MINFLUX localizations

A program was written in Python to simulate effects of fluorophore blinking behavior on MINFLUX localization. The simulations consider iterative MINFLUX localizations on a fluorophore at a given location, $\bar{r}_m$, while the fluorophore photophysical state evolution is tracked simultaneously. Firstly, the beam positions within each MINFLUX iteration are defined with a targeted coordinate pattern (TCP) consisting of 6 positions in a hexagonal grid with an additional starting position at the center (with the total number of beam positions within each TCP, $K = 7$). The diameter (L) of the TCP is varied to be 288 nm, 150m, 75nm and 40 nm for iteration 1,2,3, and 4, respectively. To keep the simulation as general as possible, without missing major features of the localization procedure which may be affected by the fluorophore blinking, the pre-localization step (iteration 0) is not simulated, given that there are several different ways of pre-localizing the fluorophore towards the center of the TCP (*6, 46*). In the simulations, we thus assumed that the fluorophore is pre-localized to be within 50nm from its actual position and that the photophysical evolution of the fluorophore due to the pre-localization step is negligible. A region of interest (ROI) of 300 nm x 300 nm was simulated with 1nm sampling, with the TCP center located at (0,0) at the start of simulation. Thus, we selected fluorophore positions within (-50, 0), (0, 50), (0, -50) and (0, 50) and considered at the onset of iteration 1, the fluorophore to be in its N (emissive *trans* isomer) state, according to

the initial condition at t=0 (Eq. S10). In the simulations we further assumed that only the N state (and not the photo-isomerized P state) is fluorescent.

During the iteration, the beam is placed at each of the beam positions for a beam dwell time, t_{dwell}. In case of pattern repeat, the pattern is repeated (within the iteration) such that t_{dwell} is the cumulative time spend at each beam position over all repeats within the TCP iteration. For example, for t_{dwell} = 150μs, pattern repeat = 1, the beam is placed at beam position i for 150 μs before it is moved to position $i+1$, whereas for t_{dwell} = 150μs, pattern repeat = 5, the beam is placed for 30μs at position i before moving to $i+1$. Once the beam has been placed on each of the K beam positions, the pattern is repeated 4 more times such that the total iteration time is the same in both cases, i.e., $t_{dwell} \times K$.

As the beam is moved from one TCP position to the next within an iteration, the intensity (and the excitation photon flux,Φ_{exc}) experienced by the fluorophore, and hence the excitation rates experienced by the fluorophore at position ($\bar{r}_m$) change. This will influence the state evolution, as described in Section S3. Here, Φ_{exc} is given by the power of the laser beam, its cross section, and how the laser beam is located with respect to the fluorophore. In the simulations, the donut size related parameter (FWHM) was set to 360 nm such that the peak-to-peak diameter is at 1.2FWHM, i.e., 432 nm (See supplementary equation S17 in reference (*5*)). The time and intensity-dependent state evolution of the fluorophore are simulated with time steps (Δt) of 100 ns. The selected Δt is long enough that equilibration between singlet ground and excited states in N and P has taken place (see main text), yet short enough to resolve the μs-ms state evolution. The photophysical state evolution was modelled as a Markovian chain (*47, 48*) based on the model obtained from the TRAST measurements, see Figure 1D, with the relative brightness of the P state set to Q = 0 (Eq. S13). Based on this model (Figure S6 and Eqs S5-S13), the individual fluorophore subject to MINFLUX localization can thus occupy the states N (bright trans isomer), P (dark cis isomer), T (dark triplet) and $\dot{R}^-$ (dark photo-reduced state) with molecule being in state N at $t = 0$.

Following the rate matrix for the photophysical transitions, as stated in Eq. S6, the stochastic matrix for this Markovian chain with the rows corresponding to the populations of the four different states of the model, N, P, T and $\dot{R}^-$, is given by

$$M = \begin{bmatrix} 1-(k'_{iso}+k'_{isc})\cdot\Delta t & k'_{iso}\cdot\Delta t & k'_{isc}\cdot\Delta t & 0 \\ (k'_{biso})\cdot\Delta t & 1-(k'_{biso}).\Delta t & 0 & 0 \\ k_T\cdot\Delta t & 0 & 1-(k_T+k_{red})\cdot\Delta t & k_{red}\cdot\Delta t \\ k_{ox}\cdot\Delta t & 0 & 0 & 1-k_{ox}\cdot\Delta t \end{bmatrix} \quad \text{(S18)}$$

where the transition rates are directly obtained from the fitting of the TRAST curves (Table S1) and defined as in Section S3 (Eqs. S6-S9). At each Δt, the row corresponding to the state occupied by the molecule at the previous time step gives the probability of transition to another state or to stay in the same state. The new state is then sampled from a multinomial distribution with the given probabilities. This is repeated for the whole iteration and the effects of changing k_{01} makes the matrix M change in each beam position since k'_{iso}, k'_{biso} and k'_{isc} are excitation dependent (Eqs. S6-S9).

Following Eq. S13 (disregarding the overall detection quantum yield of the instrument and any emission from P), the number of photons from each of the beam positions at the end of each TCP iteration is calculated by

$$n_i = \sum_{j=0}^{j=t_{dwell}/\Delta t} \Delta t k_{10} q_f S_1(t) \quad with\, t = i \times t_{dwell} + j \times \Delta t \text{ for } i \in [0,1, \dots K-1]$$

(S19)

, with the instantaneous equilibrium excited singlet state S_1 population of the N state given by

$$S_1(t) = \frac{\sigma_N \cdot \Phi_{exc}(t)}{\sigma_N \cdot \Phi_{exc}(t) + k_{10}^N} \cdot N(t) \qquad \text{(S20)}$$

Here, Φ_{exc} denotes the excitation photon flux experienced by the fluorophore within the TCP iteration.

After the calculation of n_i for K beam positions the photon counts $\bar{n} = \{n_0, n_1, \dots, n_{K-1}\}$ are used for maximum likelihood estimation (MLE) of the fluorophore position (following the same procedure as described in Balzarotti et al, Science, 2017,(*5*) supplementary section 1 and 3.1.2). In order to make inferences only on the effects of photophysics on the MINFLUX localizations, microscope and detector specific variability and background is not considered.

In order to estimate the position $\bar{r}_m$ of the emitter given the photon count vector $\bar{n}$, we find the argument which maximizes the likelihood function $\mathcal{L}\,(\bar{r}|\bar{n}) = P(\bar{n}|N, \bar{r})$ which is the conditional probability of measuring the set of photons $\bar{n}$ given the total number of photons N and the position $\bar{r}_m$, given by

$$\mathcal{L}(\bar{r}|\bar{n}) = \frac{N!}{n_0! \dots n_{K-1}!} \prod_{i=0}^{K-1} p_i(\bar{r})^{n_i} \qquad \text{(S21)}$$

Where, for the background free case, $p_i(\bar{r})$ can be calculated from

$$p_i(\bar{r}) = \frac{I_i(\bar{r})}{\sum_0^{K-1} I_j(\bar{r})} \; with\, i \in [0, 1, \dots, K-1] \; \text{(S22)}$$

Since the argument which will maximize the likelihood function will also maximize the log likelihood function, we find the position estimate by finding the argument which will maximize the log likelihood function $l(\bar{r}|\bar{n})$ given by

$$\ln \mathcal{L}(\bar{r}|\bar{n}) \propto l(\bar{r}|\bar{n}) = \sum_{i=0}^{K-1} n_i \ln p_i \qquad \text{(S23A)}$$

$$\bar{r}_m^{MLE} = argmax(l(\bar{r}|\bar{n})) \qquad \text{(S23B)}$$

The TCP center is then shifted to the new estimated location of the molecule $\bar{r}_m^{MLE}$ for the next iteration where the TCP diameter L is reduced while the laser power is ramped up in multiples of the starting power and the procedure is repeated. The power ramp for the different iterations is 1x, 2x, 4x, 6x of the starting power. For reference, at a starting power of 5 µW and with the beam dimensions as stated above the corresponding maximum intensity (in the donut ring) is 3.4 kW/cm^2. For simplicity, the time taken for the MLE calculation in a real MINFLUX localization between iterations is not considered in the simulation, since the state evolution at that time is strongly depended on the beam resting position, laser power and calculation time which is not universal.

The state evolution of an ensemble of fluorophores at position ($\bar{r}_m$) instead of a single fluorophore is simulated in a similar manner as for a single fluorophore. However, instead of multinomial sampling with probabilities given by the stochastic matrix in the single molecule case, time evolution of the state vector $\bar{A} = \left[N, P, T, \dot{R}^-\right]^T$ is such that

$$\frac{\Delta\bar{A}}{\Delta t} = M(\Delta t).\bar{A}(t) \qquad \text{(S24)}$$

Where the $M(\Delta t)$ summarizes the different transition rates from the different states and is updated based on the MINFLUX beam position as in the single molecule case. The matrix is the same as stated in Eq. S6, and is given by

$$M = \begin{bmatrix} -(k'_{iso} + k'_{isc}) & (k'_{biso}) & k_T & k_{ox} \\ k'_{iso} & -(k'_{biso}) & 0 & 0 \\ k'_{isc} & 0 & -(k_T + k_{red}) & 0 \\ 0 & 0 & k_{red} & -k_{ox} \end{bmatrix} \qquad \text{(S25)}$$

The evolved state vector after any simulation time step, Δt, is then given by

$$\bar{A}_t = e^{M\Delta t}\bar{A}_{t-1} \qquad \text{(S26)}$$

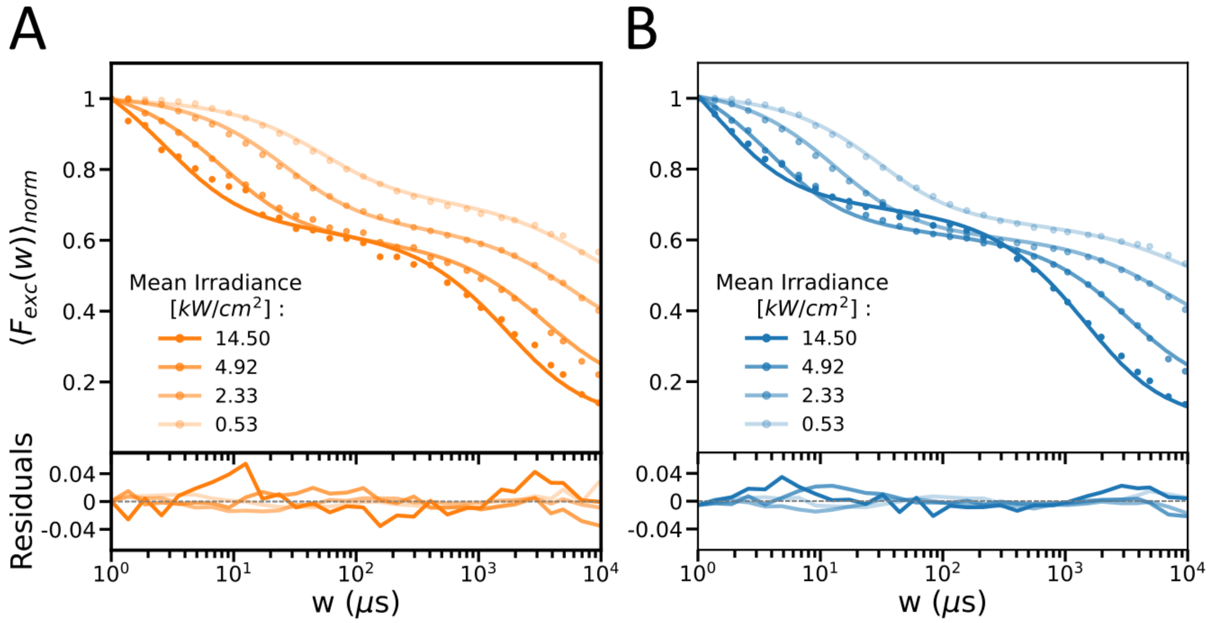

Fig. S1. TRAST curves recorded from CF750 and AF750 at different excitation intensities

TRAST curves measured from free A) CF750 and B) AF750 in PBS, with different excitation (750nm) intensities applied. Dots: experimental data, lines: fitted TRAST curves (see main text), fitting residuals below.

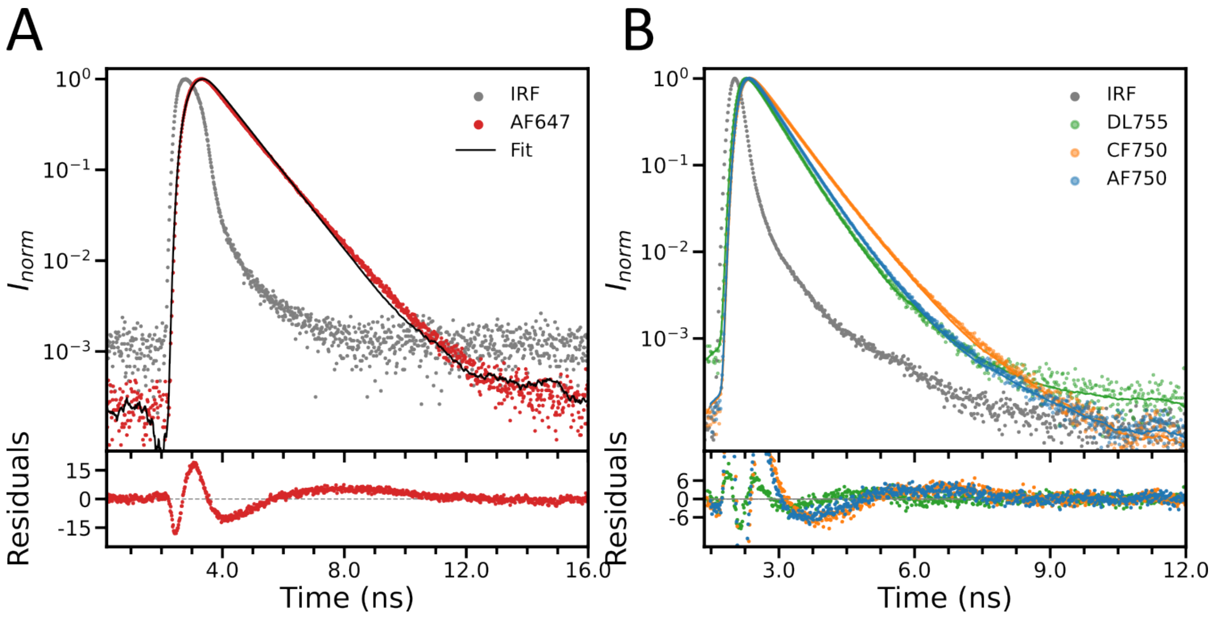

Fig. S2. Lifetime measurements for far red and NIR cyanine fluorophores

Time-correlated single photon counting (TCSPC) data from A) free AF647 with 640 nm excitation and B) NIR dyes with 750nm excitation in PBS. The fitted curve for AF647 shows a lifetime of 1.07 ns, whereas the lifetime fitted for the NIR fluorophores, DL755, CF750 and AF750, are 0.47 ns, 0.6 ns and 0.51 ns, respectively.

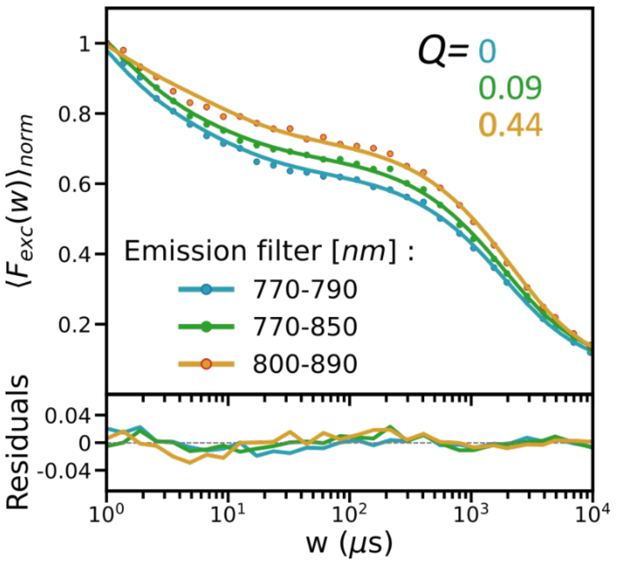

Fig. S3. TRAST curves recorded from DL755 with different emission filters

TRAST curves recorded from DL755 with different emission filters (I_exc = 4.5kW/cm2, excitation at 750nm), with experimental data represented by dots, and fitted curves with lines. Fitting residuals plotted below. The Q value represents the relative brightness of red-shifted cis (P) state emission with respect to all trans (N) state emission (Eq. S14).

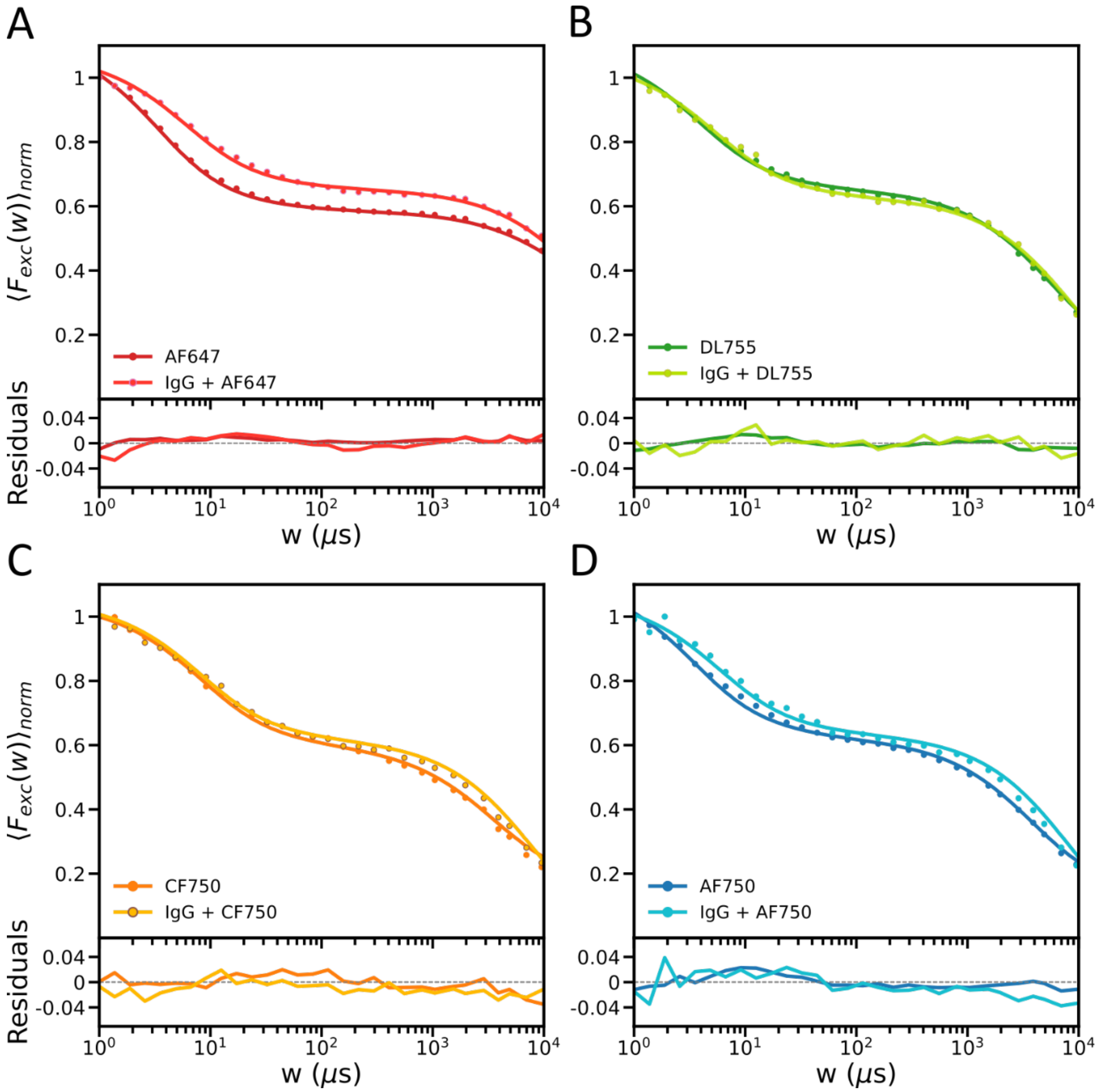

Fig. S4. Antibody labelling effect in TRAST measurements

TRAST curves recorded from A) AF647, B) DL755, C) CF750 and D) AF750, in free form and when conjugated to an antibody (IgG) All measurements for the NIR fluorophores were performed with excitation at 750nm (4.9 kW/cm2) and at 640nm (4.7 kW/cm2) for AF640.

Compared to free fluorophores, conjugation to antibodies resulted in longer isomerization relaxation times, as illustrated above. This reflects lower isomerization and back-isomerization rates (Table S1), and is in agreement with previous observations that bulky substituents on the cyanine head groups can increase the viscous drag, retarding conformational reorganizations within the molecule as well as rates for photo-induced isomerization and back-isomerization (*28, 23*).

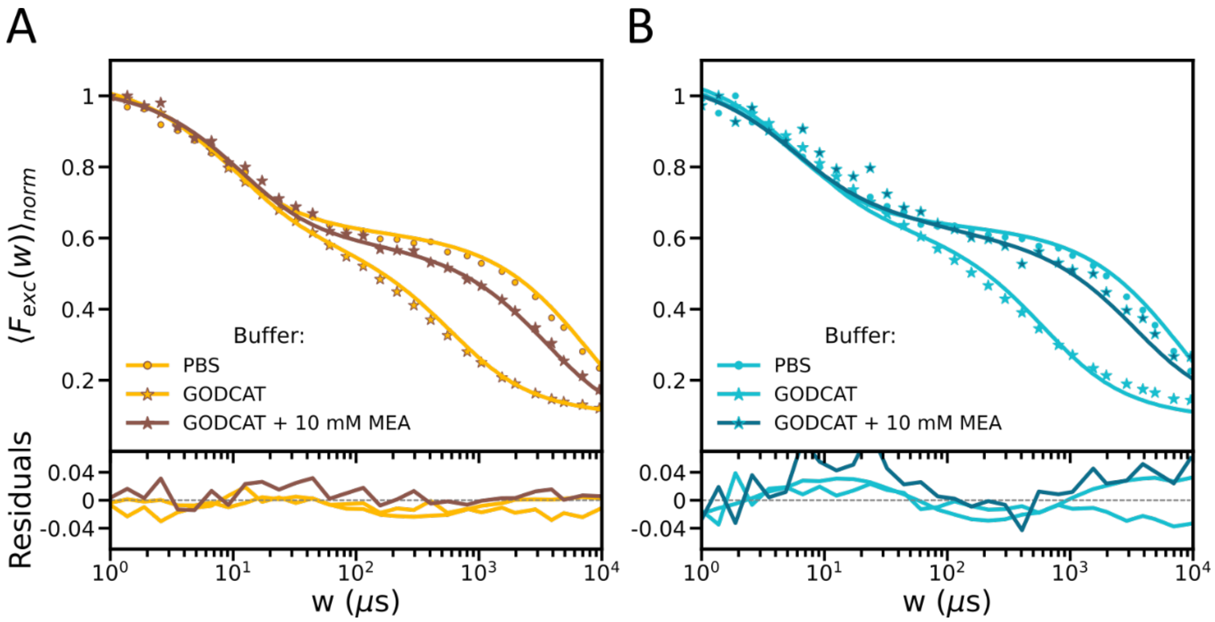

Fig. S5. Buffer effect in TRAST measurements

TRAST curves recorded from A) CF750 and B) AF750, in a Tris buffer upon deoxygenation (by adding GODCAT), and upon adding MEA. All measurements were performed with excitation at 750 nm (4.9 kW/cm2).

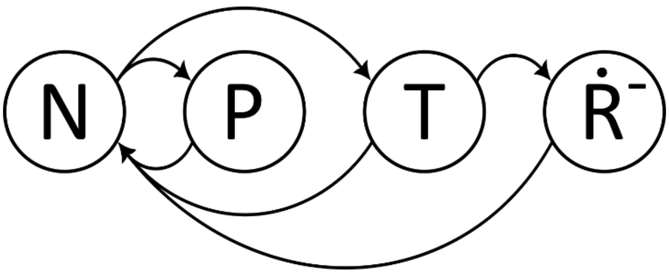

Fig. S6. Markovian chain model
Markovian chain model for the fluorophore photophysical states with the possible transitions between the states. The fluorophores undergo fluorescence blinking between the bright trans isomer state (N) and the dark cis isomer (P), triplet (T) and the long-lived redox ($\dot{R}^{-}$) state. The $\dot{R}^{-}$ state is populated through the T state, but relaxes back to the N state, following the model of Figure 1D.

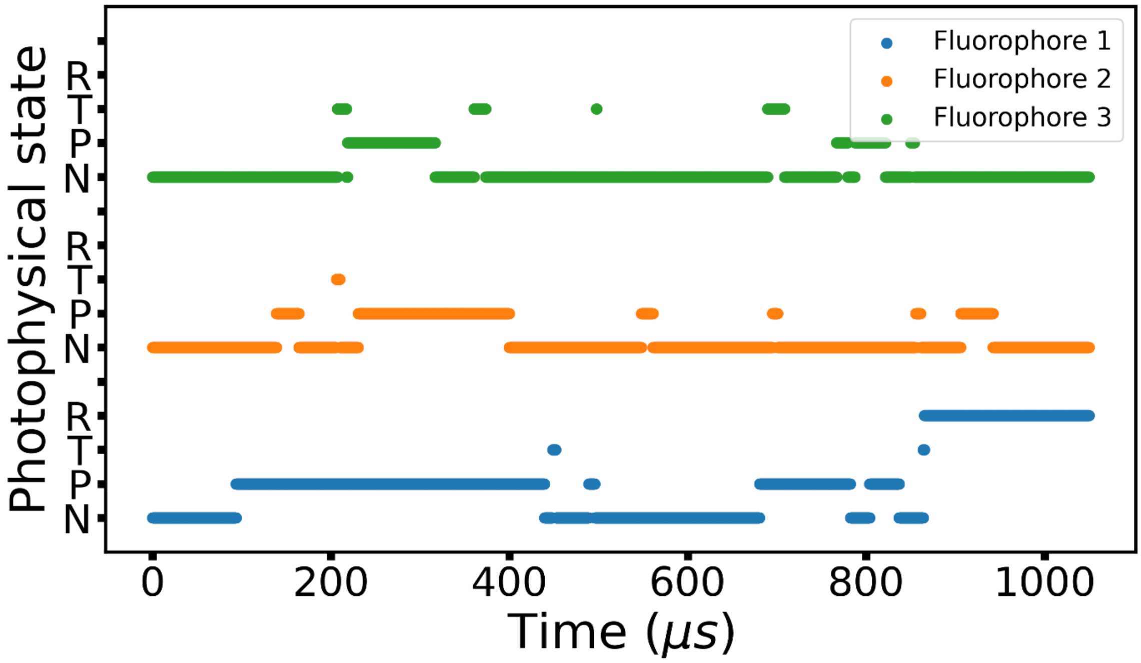

Fig. S7. Simulated single molecule photophysical state evolutions upon onset of excitation

Examples of single molecule photophysical state evolutions for three different DL755 fluorophores experiencing constant $k_{01} = 1\mu s^{-1}$. The fluorophores undergo fluorescence blinking, generated by transitions between the bright trans isomer state (N) and the dark cis isomer (P), triplet (T) and the long-lived redox (R) state, according to the model in Figure 1D and based on determined rate parameters for DL755 (Table S1).

The different implementations for the state evolution for ensemble and single fluorophores were compared to see if the ensemble evolution is recreated if many single molecule evolutions are averaged. The state evolution over ~1ms with a constant $k_{01} = 1\mu s^{-1}$ was simulated for 1000 single DL755 fluorophores (see examples above) and then averaged to compare with the ensemble case with the same conditions (Figure S8). The averaged state evolution obtained from the single molecule case, was found to agree well with the ensemble simulations.

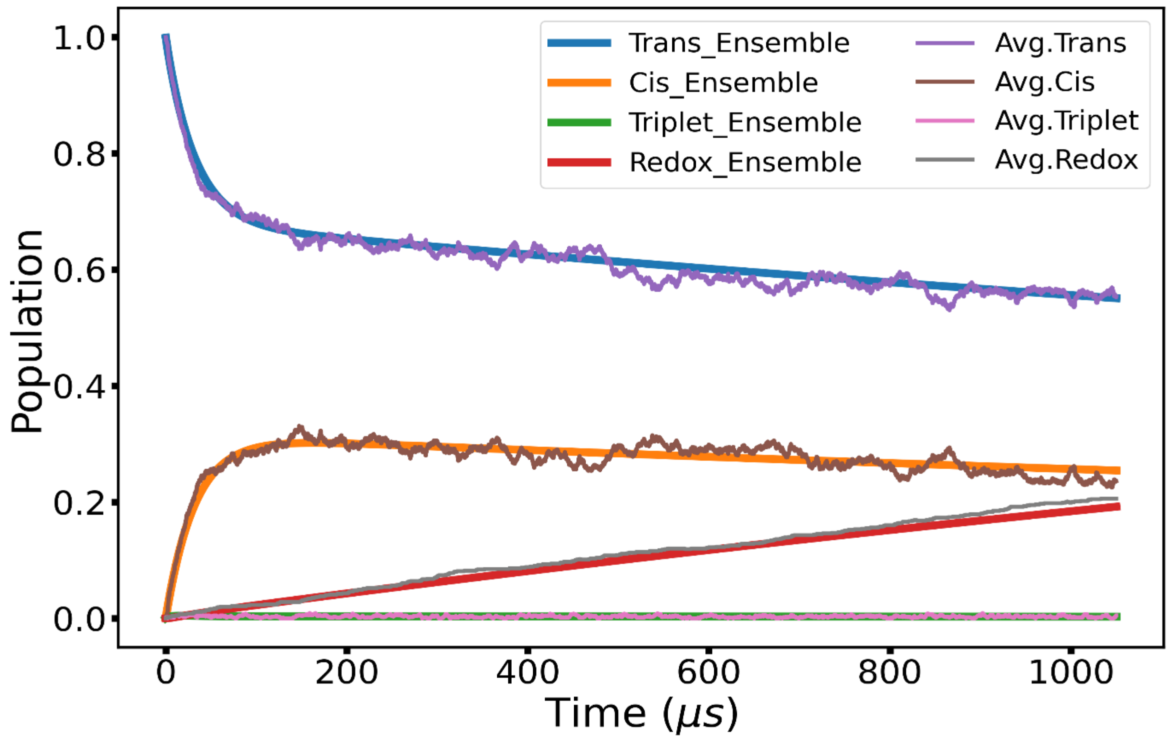

Fig. S8. Comparison between ensemble and single molecule photophysical state simulations

Comparison of simulated ensemble and averaged single molecule state evolutions of DL755 at onset of constant excitation with $k_{01} = 1\mu s^{-1}$. In the case of the single molecule simulations, the population of each state is averaged for 1000 fluorophores.

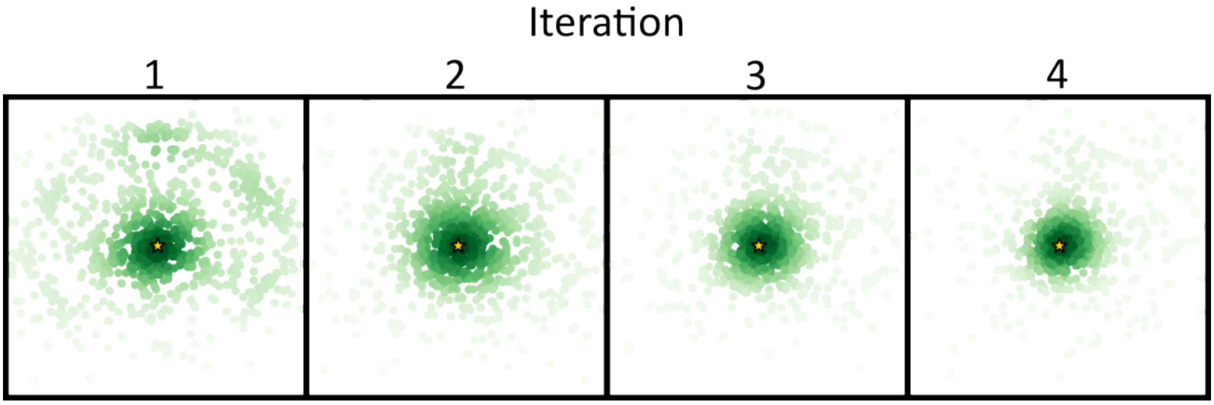

Fig. S9. Variation in localization estimates with iterations in a simulation of DL755 under conditions with lower extent of redox state transitions

Maps of estimated locations (colored dots) of a fluorophore simulated to be at position (1nm,1nm) (golden star). Estimated locations are shown at the end of each iteration of the simulated iterative MINFLUX localizations. Errors in localization and the extent of inaccurate localizations reduced with iterations (DL755 with beam dwell time of 5µs, starting laser power of 10µW and without pattern repeat). The ROI (each box) has the dimensions -100nm to +100nm in the x and y directions.

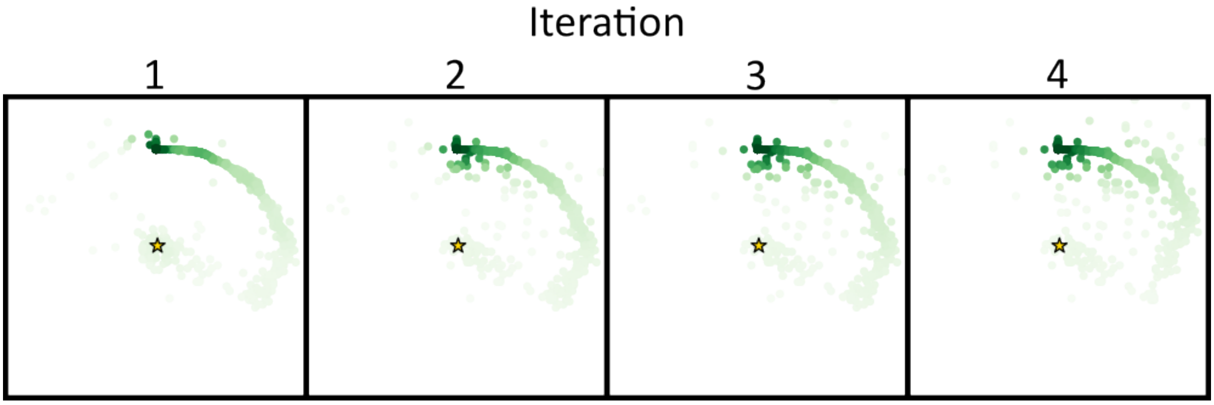

Fig. S10. Variation in localization estimates with iterations in a simulation of DL755 under conditions with higher extent of redox state transitions

Maps of estimated locations (colored dots) of fluorophore simulated to be at position (1nm,1nm) (golden star), simulated as in Figure S9, but now with beam dwell times of 150µs instead of 5µs. In this case, additional iterations do not lead to any notable improvements in the localizations. This can be attributed to the fact that in most cases the fluorophore is populated into the long-lived dark redox state during the first iteration and is not recovered until after the simulation time. In some of the simulations, in which the fluorophore came back to the N state, showed improved estimates, to an extent depending on the TCP size and position with respect to the fluorophore position. The ROI (each box) has the dimensions -100nm to +100nm in the x and y directions.

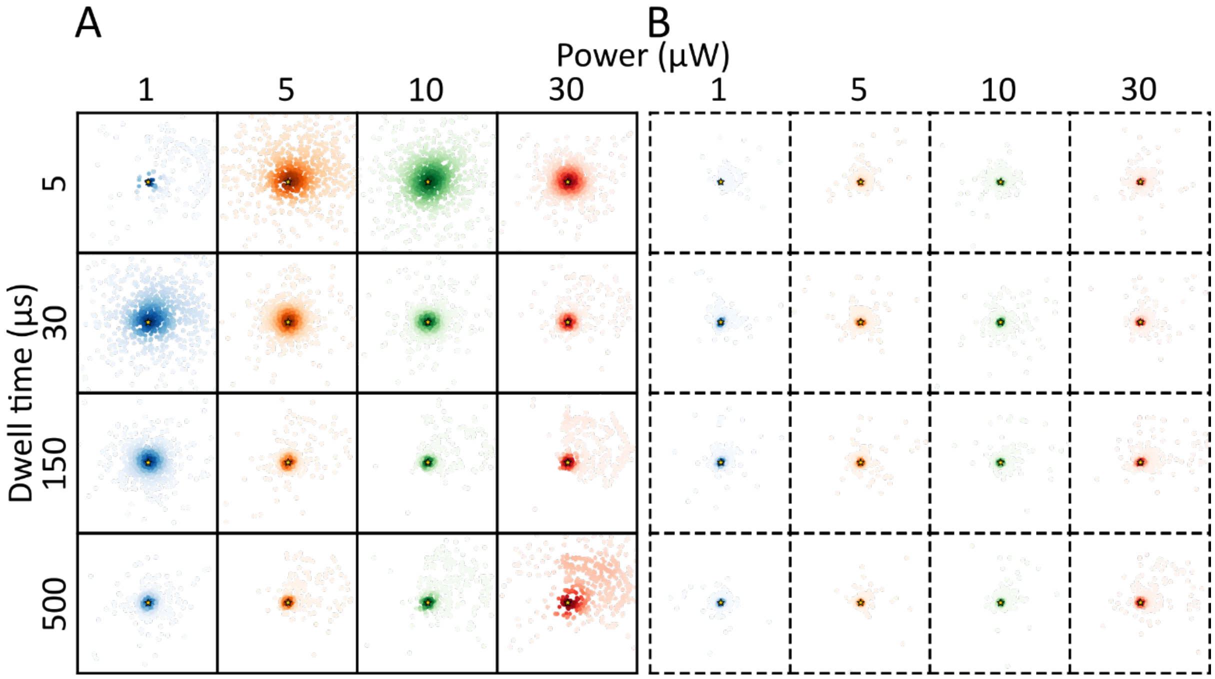

Fig. S11. Maps of estimated locations from simulations of AF647

Maps of simulated final estimated locations (colored dots) for a AF647 fluorophore at position (1nm,1nm) (golden star) after iterative MINFLUX localization. The figure shows simulated localization errors for different powers, different beam dwell times and without (A) or with (B) pattern repeats (dashed boxes, pattern repeat of 5). With pattern repeats, most of the estimates fall onto the actual position with similar localization errors for different powers and beam dwell times. The ROI (each box) has the dimensions -100nm to +100nm in the x and y directions.

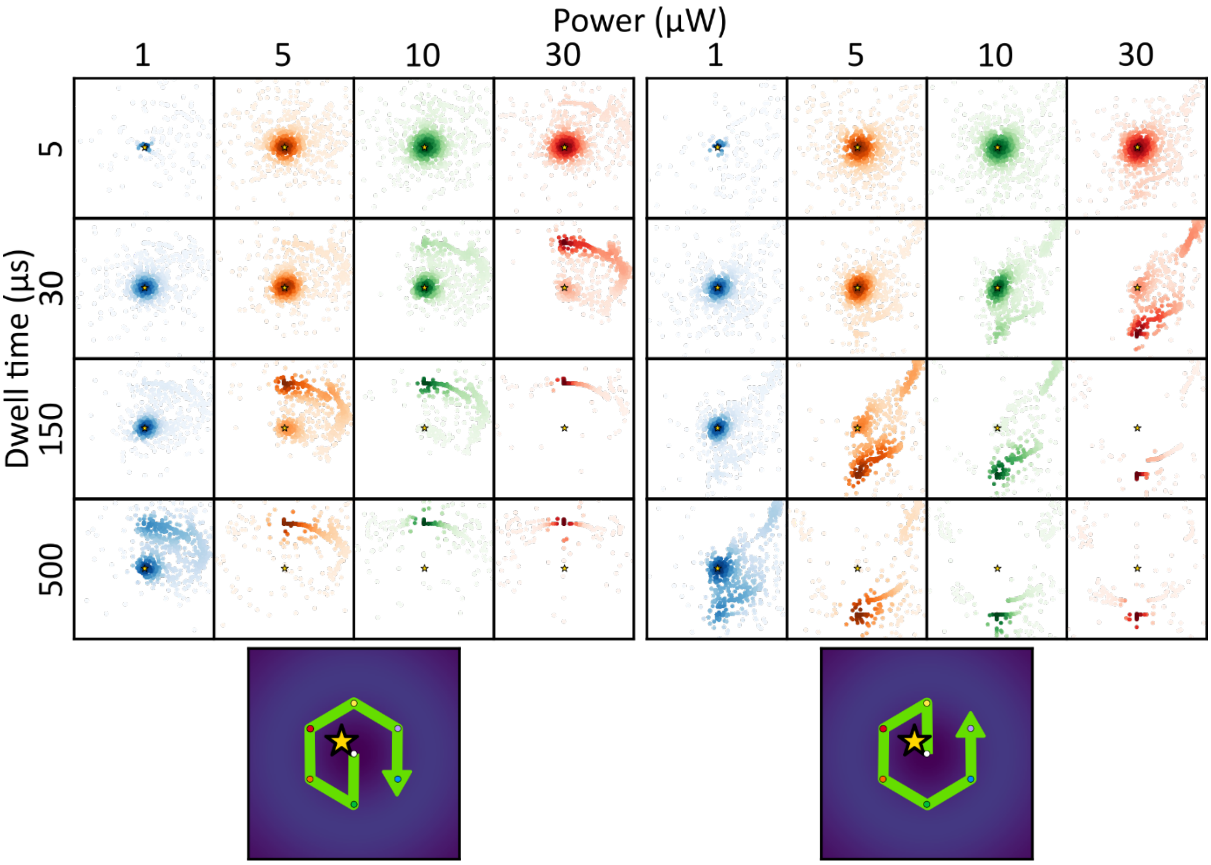

Fig. S12. Maps of estimated locations from simulations of DL755 with different TCP directions

Maps of simulated final estimated locations (colored dots) for a DL755 fluorophore at position (1nm,1nm) (golden star) after iterative MINFLUX localization. The figure shows the localization error distributions for different powers, different beam dwell times, with different beam directions. The left matrix shows the outcome when the position of the TCP beam is altered in a clockwise direction, and the right matrix for a corresponding counter-clockwise beam direction. ROI (each box) has the dimensions -100nm to +100nm in x and y directions.

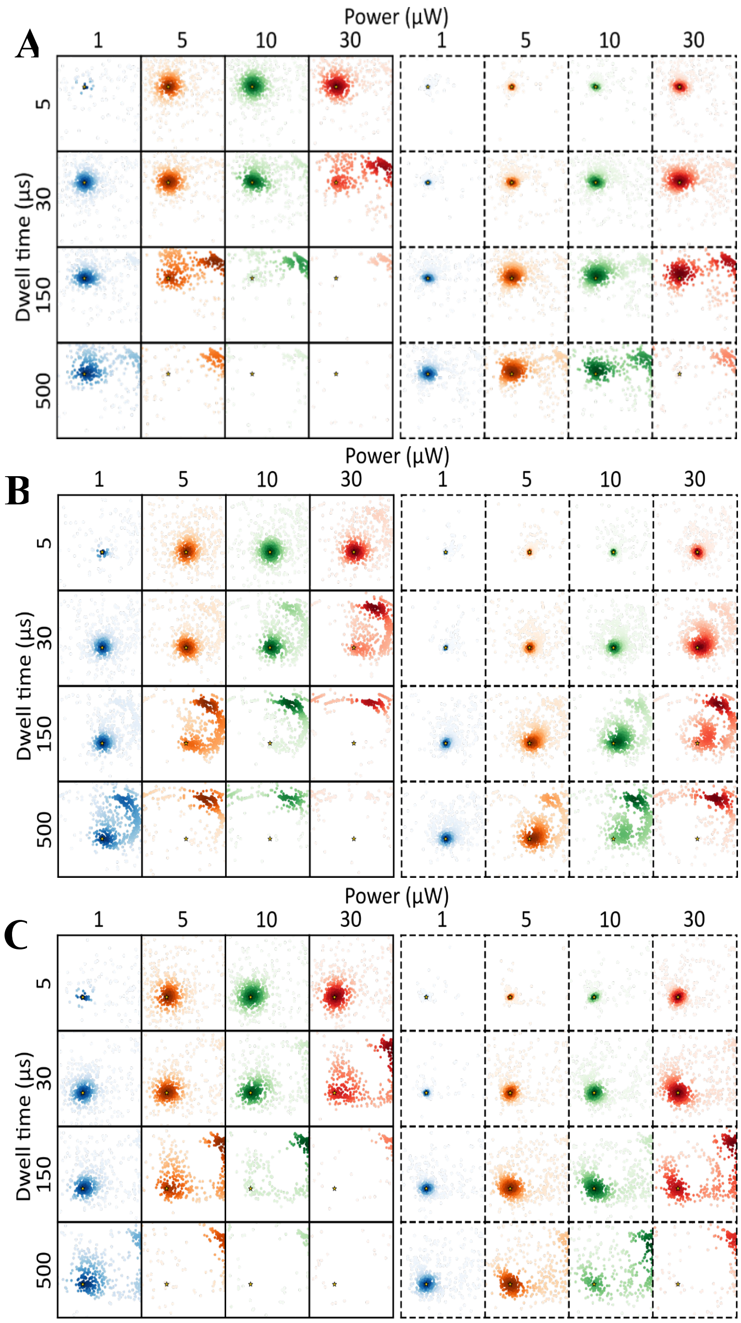

Fig. S13. Maps of estimated locations from simulations of DL755 with different fluorophore positions

Maps of simulated final estimated locations (colored dots) for a DL755 fluorophore at position (A) (-35nm,35nm) (B) (-5nm,20nm) (C) (-40nm,-30nm) (golden star) after iterative MINFLUX localization. The figure shows the localization error distributions for different powers, different beam dwell times and without (left) or with (right) pattern repeats (dashed boxes, pattern repeat of 5). The figure shows that the spatial distribution of localization errors strongly depends on the fluorophore position, clearly showing different patterns of errors with respect to Figure 3, main text. However, the overall features for how localizations are compromised by the dark state transitions (photo-isomerization, photo-reduction) remain the same. ROI (each box) has the dimensions -100nm to +100nm in the x and y directions.

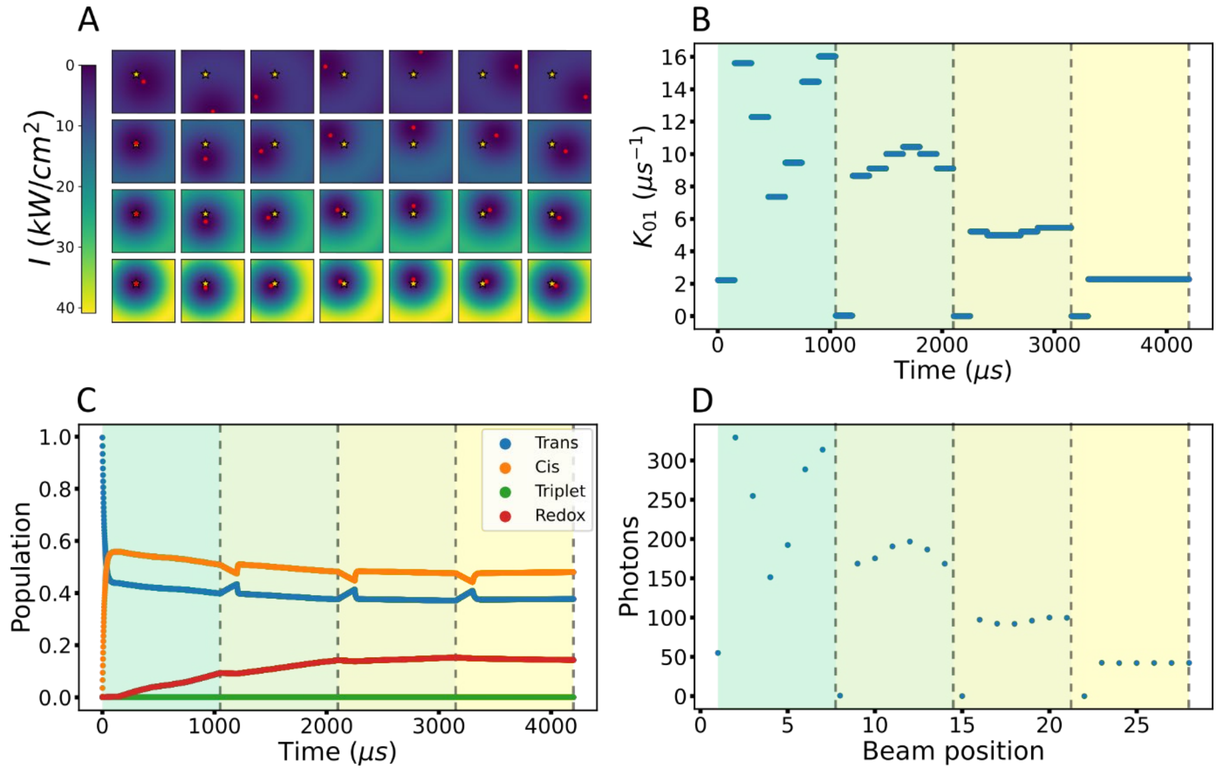

Fig. S14. Simulation of a representative MINFLUX localization for an ensemble of DL755 fluorophores

Simulation of a MINFLUX localization, based on an ensemble population kinetics, in contrast to the single molecule simulations in Figure 2 (main text). A) Illustration of the fluorophore position (golden star, in this example located at (-35nm,35nm) with respect to the TCP center in the first iteration) and the different beam positions (beam centers marked as red dots) over the different TCP iterations (corresponding to each row), with the excitation intensity increasing and the diameter of the TCP (L) decreasing from one TCP iteration to the next. B) The excitation rate (k_{01}) experienced at the fluorophore position over time as the beams are moved in the four TCPs. C) The ensemble photophysical state evolution showing the normalized populations of different states at the corresponding time (N, blue) and the dark cis isomer (P, orange), triplet (T, green) and the dark long-lived redox ($\dot{R}^{-}$, red) state during the time of localization. D) The integrated photons collected from the fluorophores for each of the beam positions shown in figure A. The different colored regions indicate different iterations in figure B, C and D. The dashed lines indicate the end of each iteration.

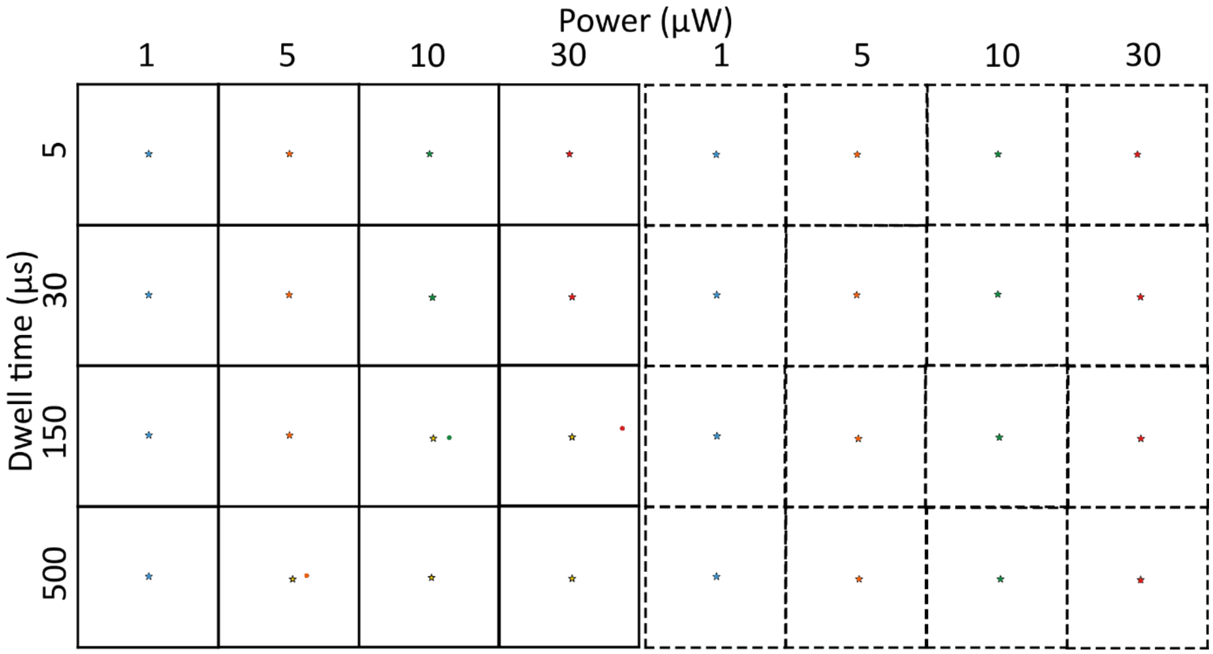

Fig. S15. Maps of estimated locations from simulations of an ensemble of DL755

Maps of simulated final estimated locations (coloured dots) for an ensemble of DL755 fluorophores at position (1nm,1nm) (golden star) after iterative MINFLUX localization. The figure shows simulated localization errors for different powers, different beam dwell times and without (A) or with (B) pattern repeats (dotted boxes, pattern repeat of 5). With pattern repeats, all the estimates fall onto the actual position for different powers and beam dwell times. The ROI (each box) has the dimensions -100nm to +100nm in the x and y directions.

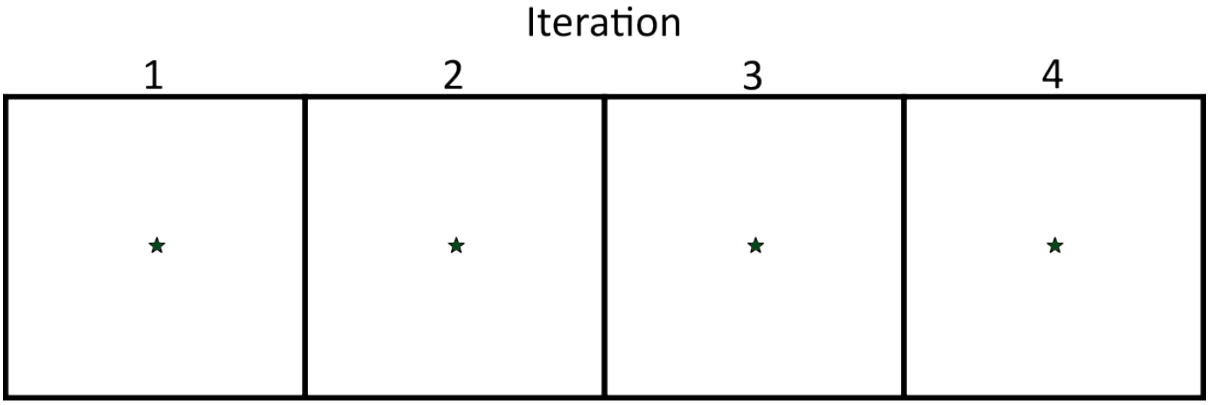

Fig. S16. Simulations of localization estimates of a DL755-like fluorophore with no dark state transitions with iterations

The map of simulated estimated locations (colored dots) of a DL755 like fluorophores, with no blinking due to isomerization or photo-reduction, at position (1nm,1nm) (golden star) at the end of each iteration of the simulated iterative MINFLUX localization. In the absence of blinking and any background, the simulation can accurately localize the single molecule fluorophores. The ROI (each box) has the dimensions -100nm to +100nm in the x and y directions.

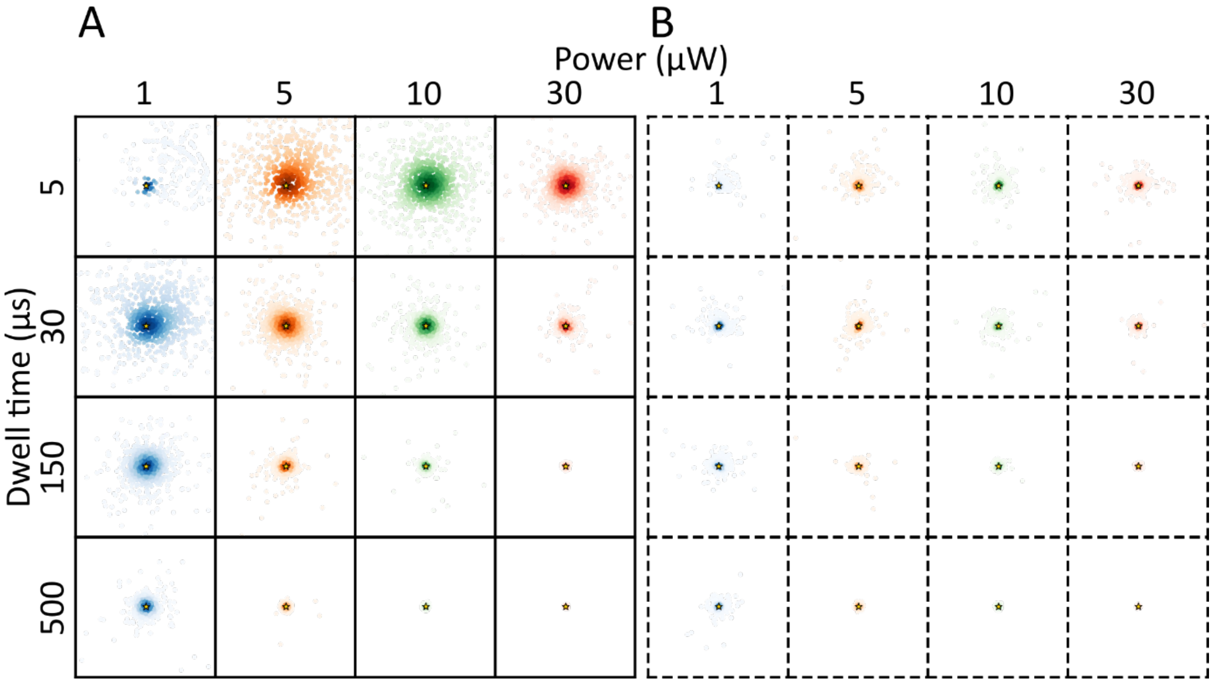

Fig. S17. Maps of estimated locations from simulations of a hypothetical AF647 like fluorophore, with no long-lived redox state

Maps of simulated, final estimated locations (colored dots) for a AF647-like fluorophore, with no long-lived redox state, at position (1nm,1nm) (golden star) after iterative MINFLUX localization. The figure shows the localization errors for different powers, different beam dwell times, and without (A) and with (B) pattern repeats (dashed boxes, pattern repeat of 5). With pattern repeats, most of the estimates are accurately localizing the actual fluorophore position with similar localization errors between different powers and beam dwell times. The ROI (each box) has the dimensions -100nm to +100nm in the x and y directions.

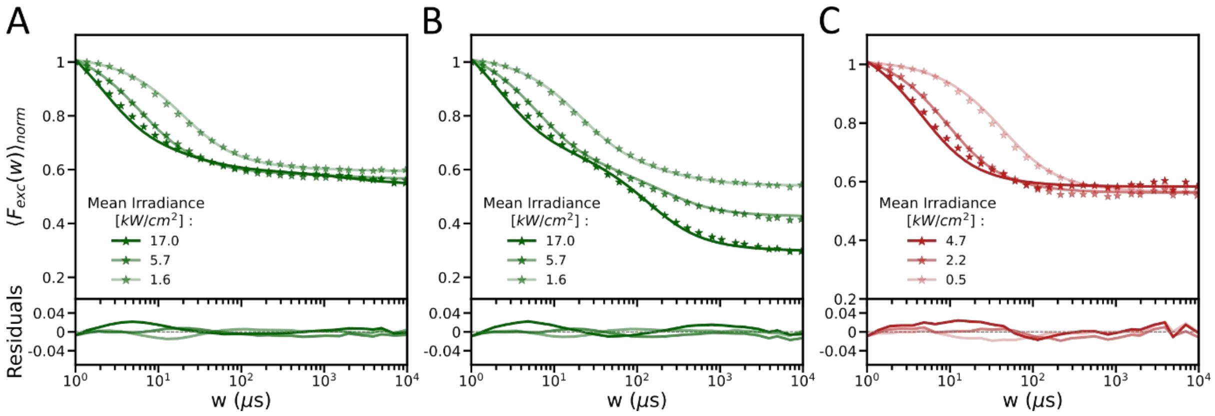

Fig. S18. ROXS effect in TRAST measurements

TRAST curves with different excitation intensities applied, from (A) DL755 recorded in TAE buffer with addition of GODCAT and ROXS (1mM MV and 1mM AA), (B) DL755 in Trolox buffer (TAE + GODCAT + 5mM Trolox) and (c) AF647 recorded in Redox buffer (TAE + GODCAT + 1mM MV + 1mM AA)

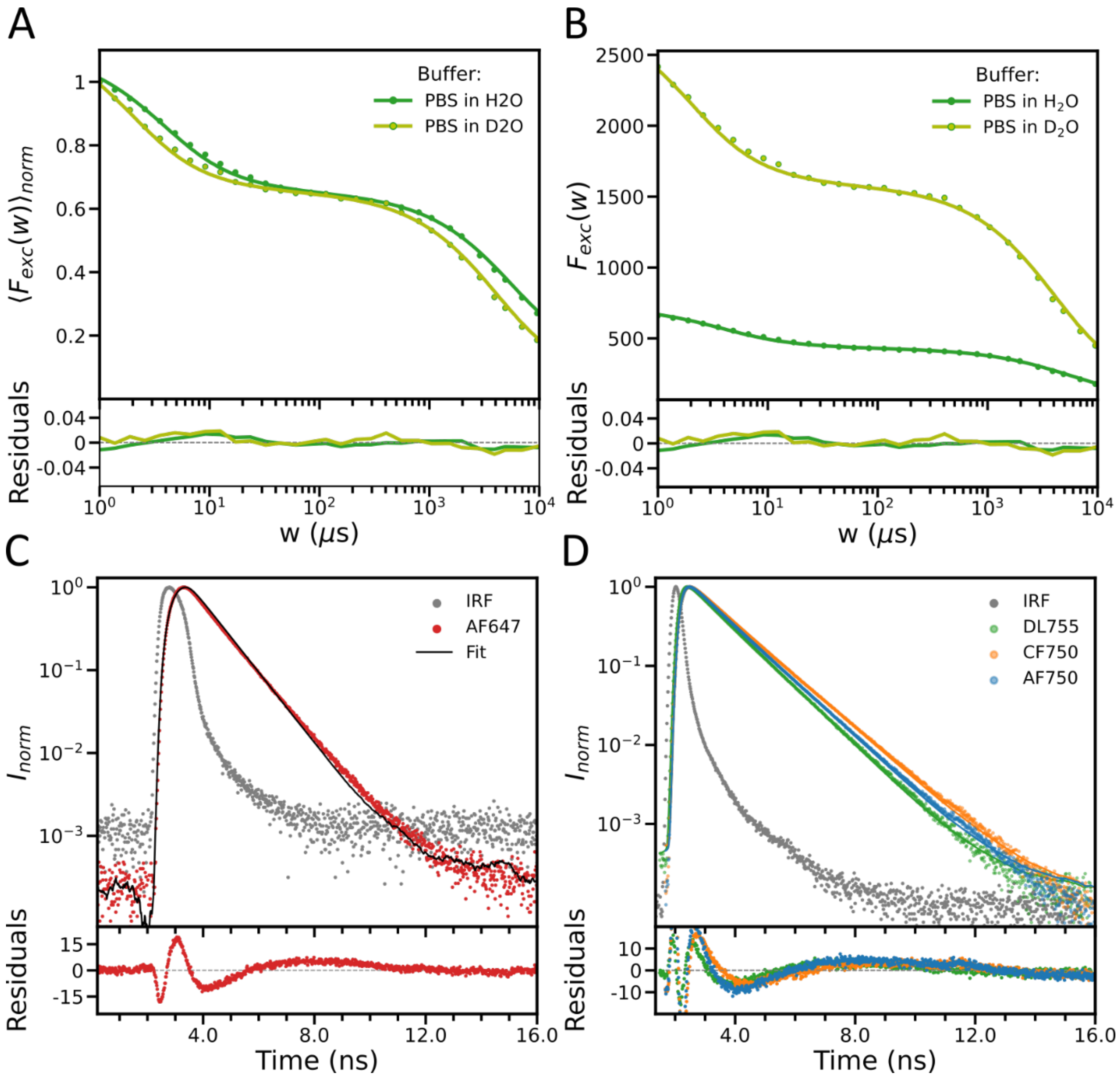

Fig. S19. Heavy water effect in TRAST and lifetime measurements

A) Normalized and B) non-normalized TRAST curves from DL755 in PBS using H_2O or D_2O as the solvent (Excitation at 750nm, with 4.9 kW/cm). TCSPC measurements from free C) AF647 and D) NIR dyes in D_2O. The fitted curve for AF647 shows a lifetime of 1.2 ns, compared to 1.07ns in H_2O (Figure S2A). The lifetimes recorded for DL755, CF750 and AF750 fitted to 1.1, 1.3 and 1.2 ns, respectively, to be compared to 0.47ns, 0.6ns and 0,51ns, as obtained in H_2O (Figure S2B).

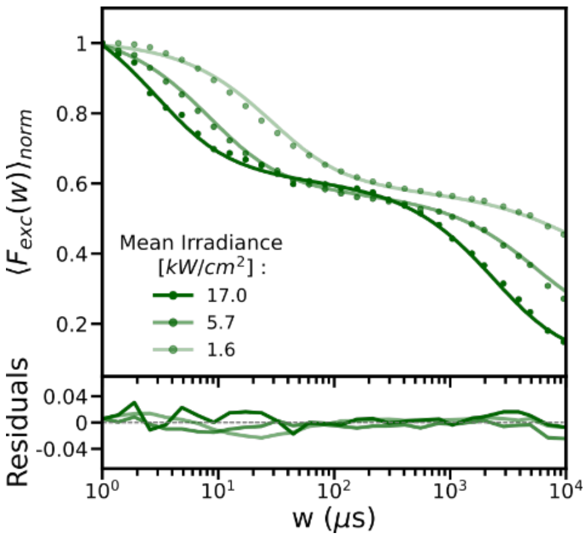

Fig. S20. Excitation intensity dependence in TRAST curves recorded from DL755 in a commercial imaging buffer

TRAST curve from DL755 conjugated to F1 imager strands recorded in an imaging buffer from Massive Photonics with different excitation intensities applied.

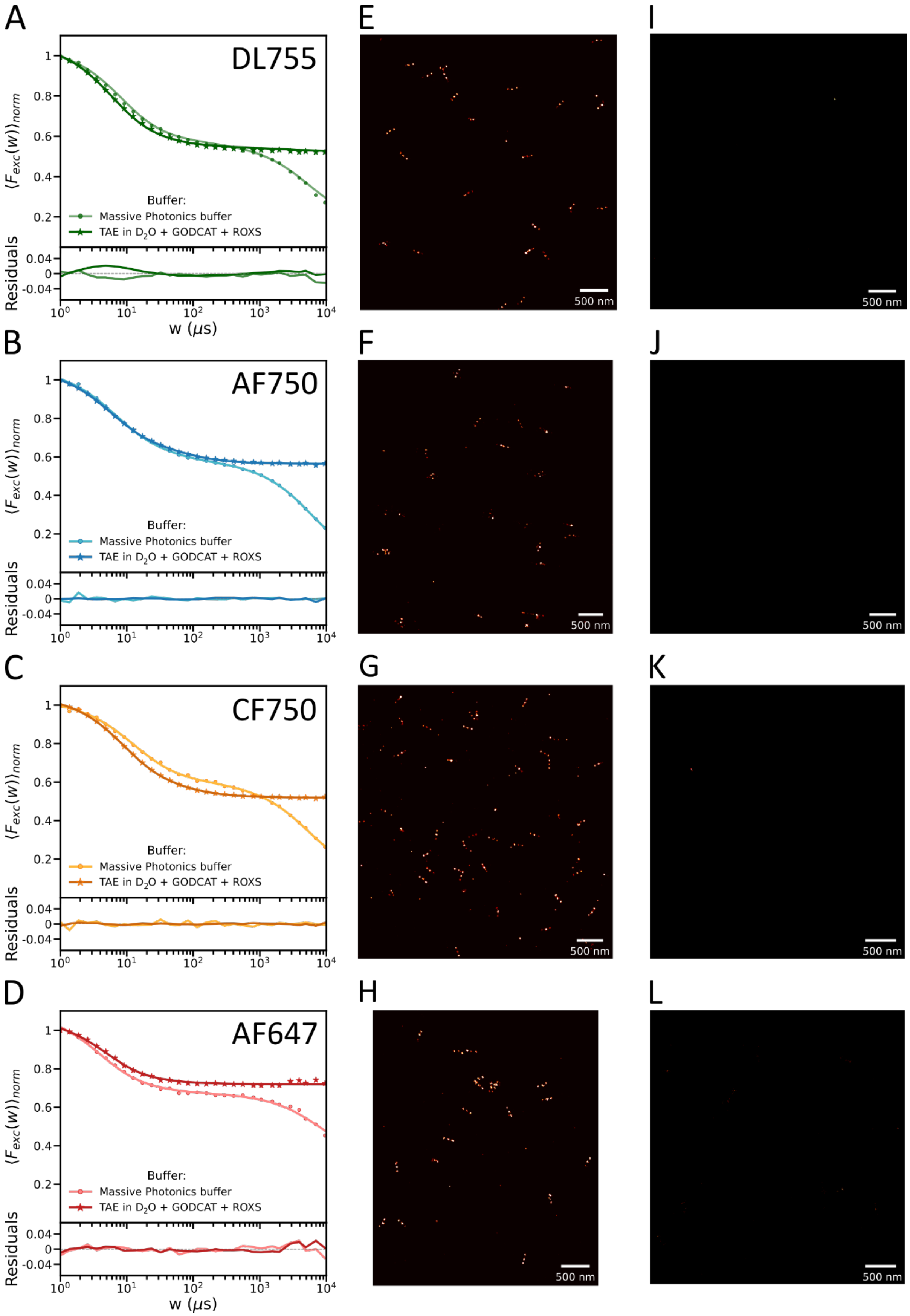

Fig. S21. TRAST curves (A-D) and MINFLUX images (F-H and I-L) recorded in different redox buffers

(A-D): TRAST curves recorded from A) DL755 (750 nm, excitation intensity, I_{exc}= 5.7 kW/cm^2), B) AF750 (750 nm, I_{exc}= 4.9 kW/cm^2), C) CF750 (750 nm, I_{exc}= 4.9 kW/cm^2) and D) AF647 (640 nm, I_{exc}= 9.9 kW/cm^2), all conjugated with F1 strands, in a commercially available imaging buffer from Massive Photonics or in a deuterated redox-balanced buffer (TAE in D_20, GODCAT and ROXS, described in Material and Methods).Use of the balanced redox buffer efficiently suppresses $\dot{R}^-$ state buildup in the fluorophores.
(E-H): DNA PAINT MINFLUX images of nanorulers (see Materials and Methods), using F1 imager strands conjugated with E) DL755 F) AF750, G) CF750 and H) AF647 recorded in the deuterated redox-balanced buffer (TAE in D_20, GODCAT and ROXS).
(I-L): DNA PAINT MINFLUX images of the same nanorulers as in (E-H), now recorded in a commercial Massive Photonics imaging buffer, using F1 imager strands conjugated with I) DL755 J) AF750, K) CF750 and L) AF647.

Comparing MINFLUX images recorded with (E-H) or without a balanced redox buffer (I-L), it can be seen that without a balanced redox buffer, efficiently suppressing $\dot{R}^-$ state buildup in the fluorophores (A-D), practically no valid localizations are obtained. This is consistent with our simulation results (Fig. 3), and that inaccurate localizations are discarded during the MINFLUX imaging experiments.

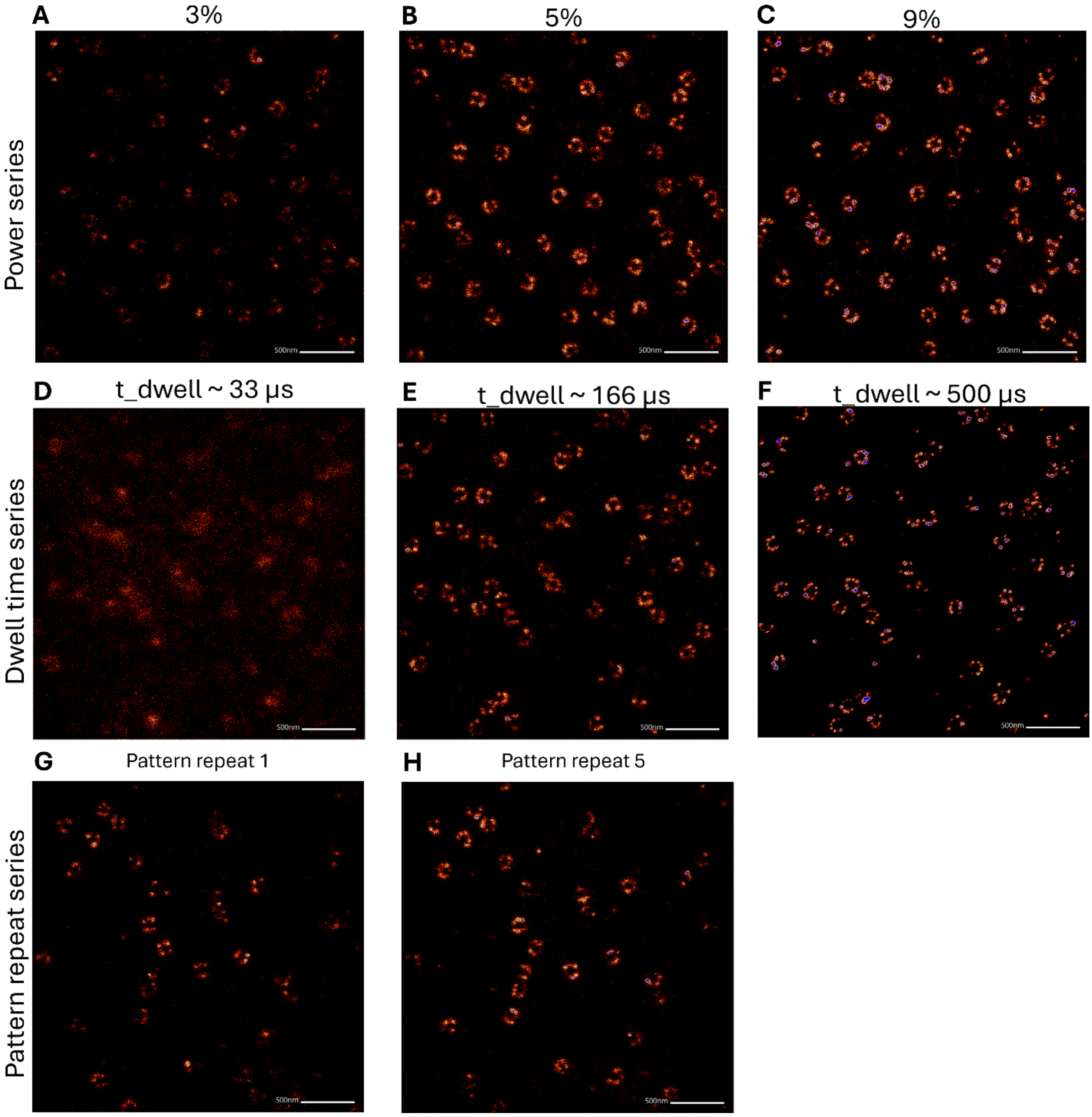

Fig. S22. DNA-PAINT MINFLUX images of NUPs recorded under different imaging conditions

Comparison of DNA-PAINT MINFLUX images of NUPs in U2OS cells (with F1 DL755 as imager strand, transiently binding to docking strands decorating the NUP96-mEGFP through an antiGFP nanobody) recorded with different excitation powers, beam dwell times, and with or without a pattern repeat (=5). All samples were kept in a deuterated balanced redox buffer (the ROXS redox buffer, see Materials and Methods). The images, within each of the parameter series, were taken at the same 3 µm x 3 µm ROI showing the same set of nuclear pore complexes and for 1 hour. The colorbar is set from 0 to 5 for all images. Relevant imaging parameters including median localization precision in x and y directions (S_x & S_y) and background frequence (FBG), were obtained by analysing the data with the pyMINFLUX software (*45*) and are listed in Table S2.

(A-C): MINFLUX images acquired with the starting power of the laser set to A) 3%, B) 5% and C) 9% (beam dwell time 166 µs, no pattern repeat). With increasing excitation powers

applied, and analyzing the acquired data with pyMINFLUX,(*45*) we observed that the localization precision improved (seen as decreased standard deviations in the localizations in the x- and y-directions, S_x & S_y), and both EFO and FBG increased. Also, the number of valid localizations increased, indicating a lowered extent of discarded localizations with higher starting powers of the laser (summarized in Table S2).

(D-F): MINFLUX images acquired with different beam dwell times: D) 33 µs, E) 166 µs, F) 500 µs. Starting power was set to 5%, with no pattern repeat. Analyzing the images in the same way as in (A-C), we found improved localization precisions when longer dwell times were implemented in the MINFLUX experiments, while EFO and FBG stayed relatively constant (Table S2). Constant EFO and FBG are expected since the starting laser power was kept the same for the different dwell times applied.

(G-H): MINFLUX images acquired without (G) and with (H) a pattern repeat (5 repeats) implemented in the imaging sequence. The beam dwell time was set to 166 µs (i.e. 166 µs on each beam position in the TCP (G), or 33 µs on each position visited five times (H)). The starting power was set to 5%. Consistent with the simulations (Fig. 5), we did not observe much difference with and without pattern repeat, indicating that the localization precision is already improved without pattern repeats, with most of the photophysical effects on the localization precision managed with the buffer used (Table S2). We did not choose dwell times lower than 166 µs with pattern repeat since individual measurement times at each beam positions will then be similar to the beam travel time between the TCP positions (cannot be faster than the switching time (~ some microseconds) of the EODs in the setup).

In these experimental studies of the effects on excitation power, dwell times and pattern repeats on the MINFLUX images, it should be noted that lower photon numbers were collected (using a limit of 10 photons per iteration, see note regarding imaging software settings below). This makes the overall images and the localization precisions worse than for standard imaging sequences, as used e.g. in the images in Figure 6 (see also Table S2 and Table S4). This is however not the purpose of these measurements and images acquired (Figs S22A-H above), but rather to experimentally verify trends in the localization precision as found from the simulations, for fluorophores in a balanced redox buffer undergoing minimal redox blinking. These measurements support the outcome of our simulations, that higher excitation powers (and higher numbers of detected photons per fluorophore to be localized) and longer dwell times can improve localization.

Settings of the imaging sequences in the Imspector software (from Abberior Instruments):
For the images A-H acquired above, starting powers could be easily changed from the imaging software (Imspector) menu, while pattern dwell times and pattern repeats had to be changed in the sequence file itself. Abberior sequences have a parameter called '*patDwellTime*' which defines the time it takes to complete one full circle around the TCP (without the center position) and the minimum time spent in photon acquisition. This is slightly different from the nomenclature used in our main text where the dwell times (t_{dwell}) are defined as the time the laser beam spends at each position along the TCP. For example, setting *patDwellTime* = 0.001 seconds in the Abberior sequence file with a hexagonal pattern leads to t_{dwell} ~166 µs at each of the six beam positions on the TCP (in case of no pattern repeat). This is close to the condition t_{dwell} =150 µs, which we simulated. Pattern repeats were implemented by the *patRepeat* parameter, such that for *patRepeat*=5 and *patDwellTime* = 0.001 seconds (or t_{dwell} ~166 µs) the beam is moved in the TCP five times, with each beam position visited roughly 33 µs at each repeat. There is also the parameter '*phtLimit*' defining the minimum number of photons required to make a localization estimate and to proceed with the next iteration. In the standard imaging sequence (named '*Imaging_2D*'), '*phtLimit*' is typically set to 100-150 photons per

iteration. Within an iteration, a localization estimate and a change in the pattern size (reduction of the diameter L of the TCP) to go to a next iteration are only implemented when the minimum photon number set by '*phtLimit*' is obtained. This means that the system repeats the same motion (TCP) multiple times (multiple dwells, with the dwell time set by *patDwellTime*) until *phtLimit* is reached. To avoid this, and to evaluate differences in localizations with different powers and beam dwell times (and compare them to the trends seen in the simulations presented in Figure 5), while keeping other settings the same, we had to use a sequence with low photon limits for each iteration. We thus used a custom sequence '*seqDefault_10pht*' (Table S5) with a *phtLimit* set to 10 photons per iteration, within which we then changed the different *patDwelltime* and *patRepeat* parameters. Although the lower photon numbers resulted in lowered localization precision (Table S2), compared to that obtained from standard sequence (Table S4), as used when acquiring the images in Figure 6, the custom sequence allowed us to experimentally observe the trends seen in the simulations (Figure 5). The low photon limit of 10 photons was also not enough to keep the number of dwells per iteration to 1 for the shortest dwell time investigated (33 µs), but the resulting images are nonetheless illustrative of the changes with different pattern dwell times.

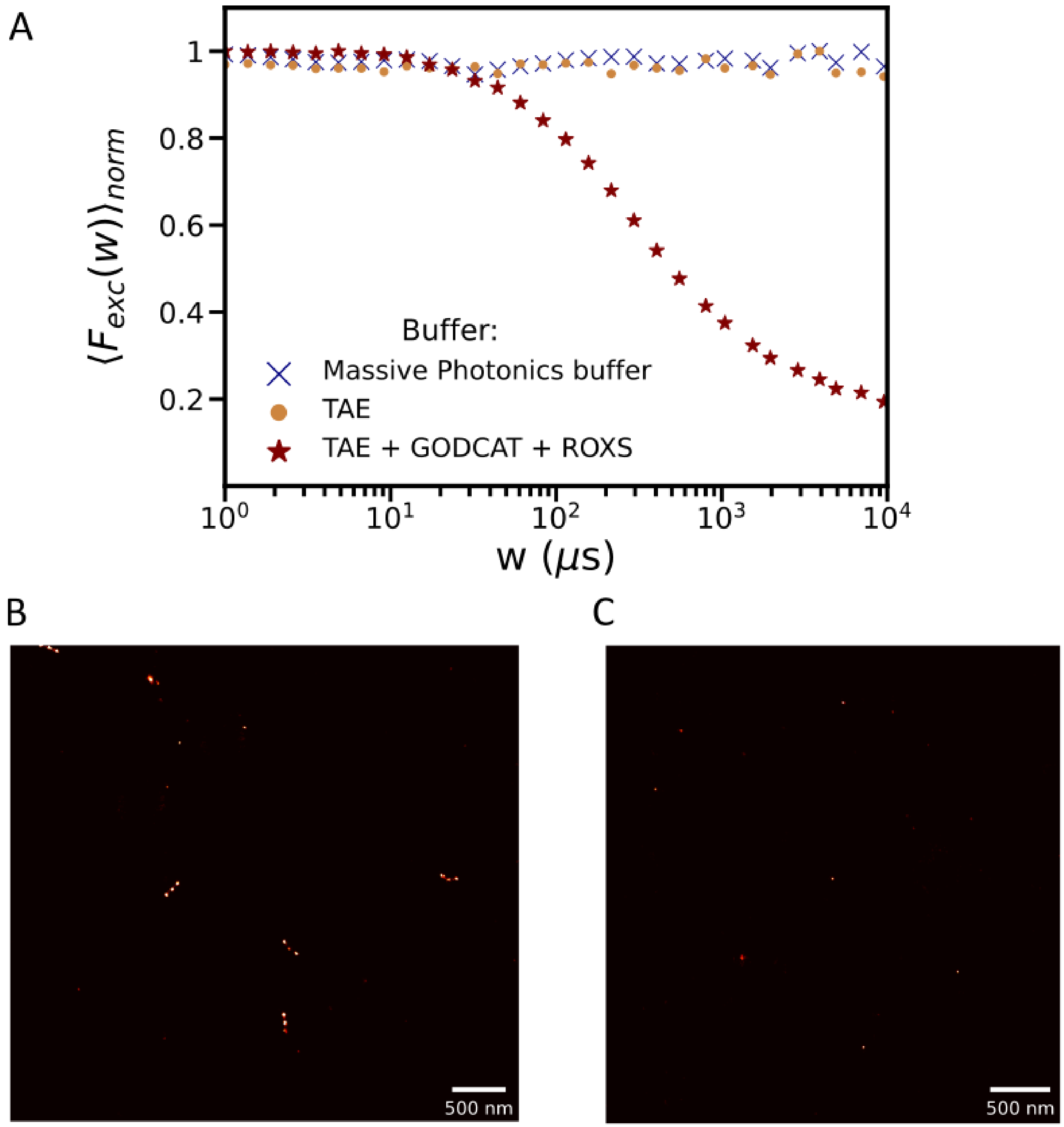

Fig. S23. TRAST measurements and DNA PAINT MINFLUX images with ATTO 700 in two different imaging buffers

A) TRAST curves from Atto700 recorded in different imaging buffers, excited with 640nm (I_{exc} = 9.9kW/cm^2). DNA PAINT MINFLUX images of nanorulers imaged with F1 imager strands conjugated with Atto700 recorded in B) a commercially available DNA-PAINT buffer from Massive Photonics, and in C) a ROXS redox buffer (TAE, GODCAT and ROXS).

A TIRF based DNA-PAINT (ROXS buffer) **B** TIRF based DNA-PAINT (MP imaging buffer)

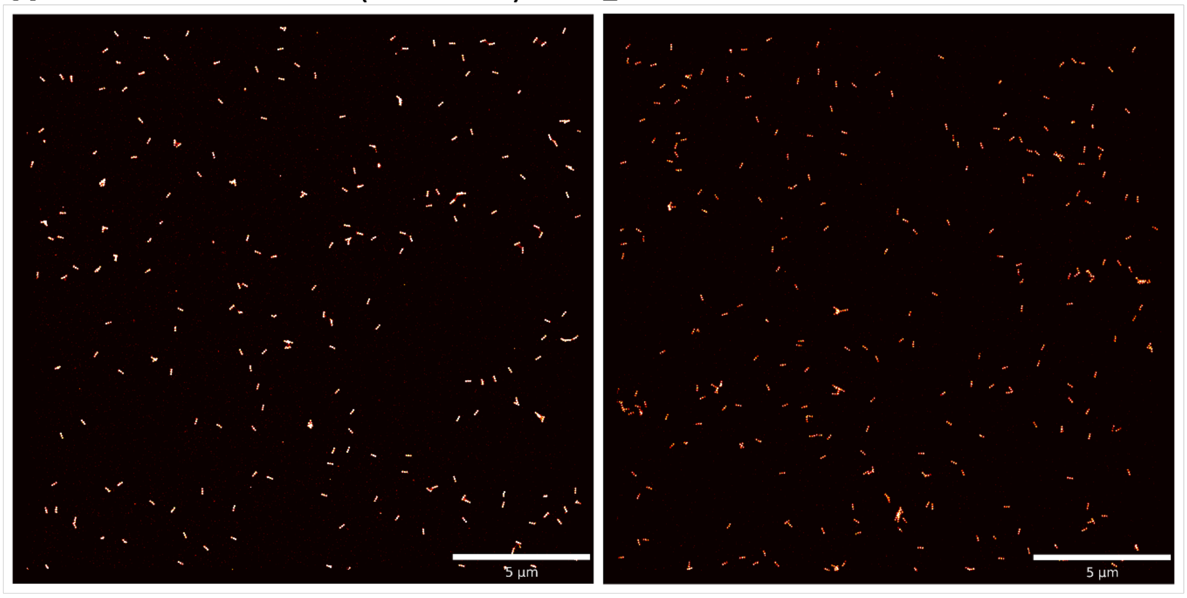

C

	DNA-PAINT MINFLUX (ROXS buffer)	TIRF based DNA-PAINT (ROXS buffer)	TIRF based DNA-PAINT (MP imaging buffer)
Loc. Precision (nm)	7.2	8	11
Events per minute	16	4036	1985
Mean photon no. per event	~4000	~12000	~1000

D DNA-PAINT-MINFLUX (ROXS buffer)

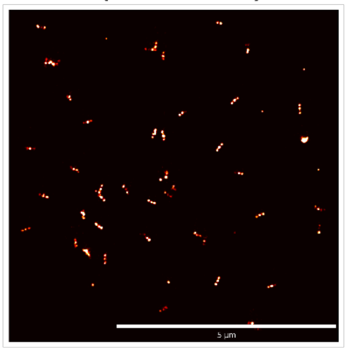

Figure S24. DNA-PAINT and DNA-PAINT MINFLUX images of nanorulers
(**A, B**): Comparison of NIR (TIRF based) DNA PAINT images of GATTAquant nanorulers imaged with DL755 imager strands in A) deuterated redox balanced (ROXS) buffer and in B) commercially available (Massive Photonics) imaging buffer. **(C):** The average number of photons per binding event and the localization precision improved when using the redox balanced buffer compared to the commercial buffer, owing to suppression of fluorophore blinking. The improved localization precision is comparable to that observed for NIR DNA-PAINT MINFLUX images with the same sample, shown in (**D**). While the number of events detected (correlating to imaging speed) are higher with parallelized TIRF-based DNA PAINT, MINFLUX imaging achieves the same localization precision with much lower number of photons (~650 photons per localization in this investigation).
The DNA PAINT experiments were performed on a total internal reflection fluorescence (TIRF) microscope, as described in, (*42*) with addition of a 750 nm laser for excitation, and associated dichroic and emission filters. The GATTAquant nanorulers samples were prepared following the protocol reported in Materials and Methods (main text).

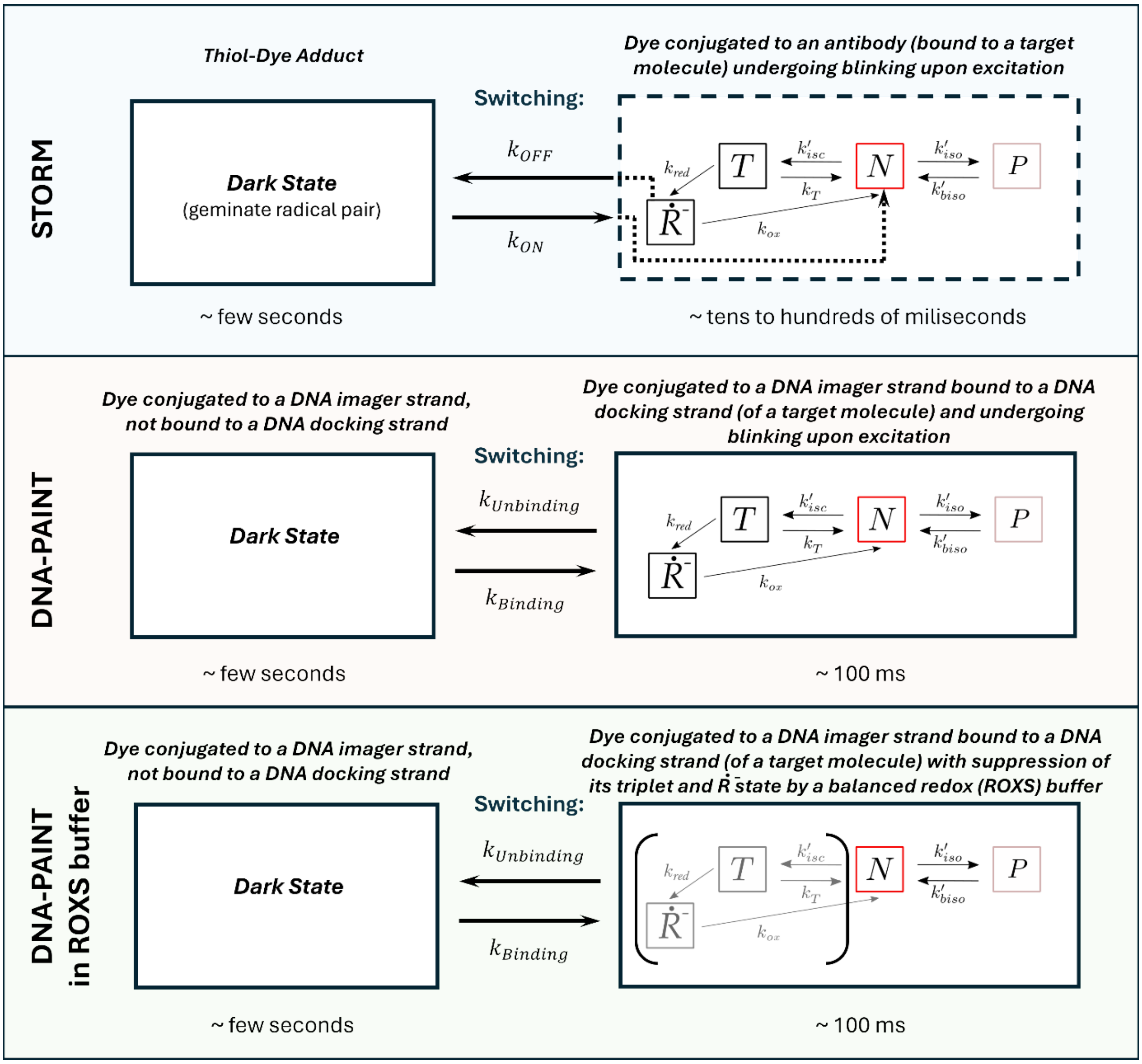

Fig. S25. Overview of switching and blinking mechanisms for the cyanine fluorophores under the different labeling and buffer conditions investigated in this study.

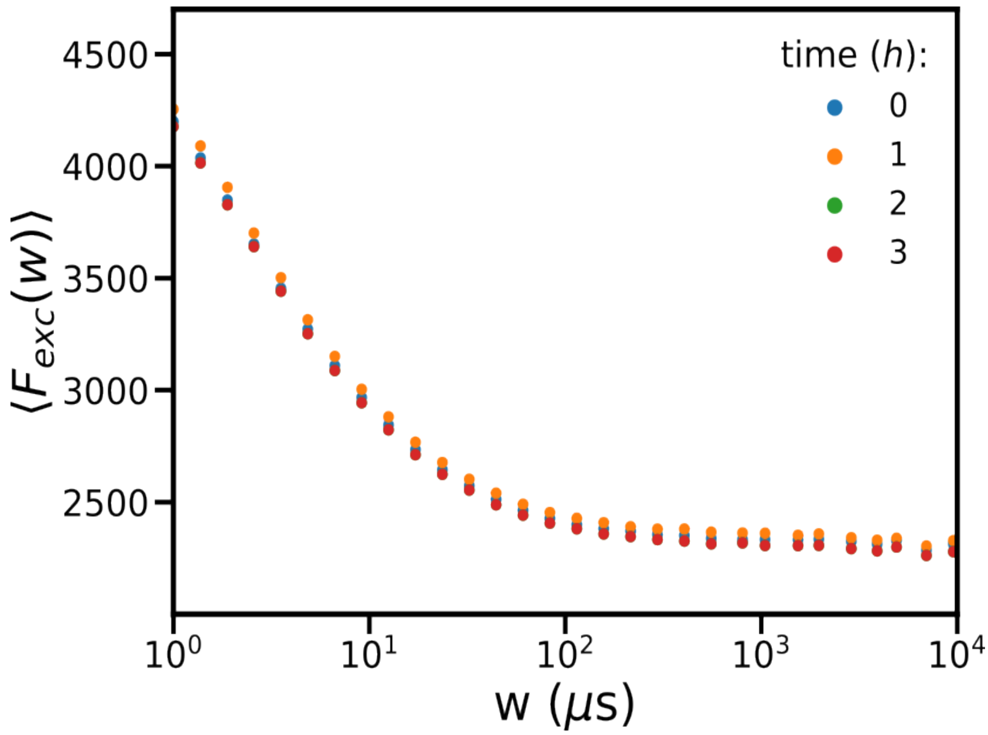

Fig. S26. TRAST curves recorded from DL755 in a redox balanced buffer under air-saturated conditions

Non-normalized TRAST curves from DL755 conjugated with F1 imager strand in a deuterated redox buffer (TAE in D_20, GODCAT and ROXS), placed in a non-sealed well chamber, open to air exchange, and recorded at 0 to 3 hours after application of the sample in the well chamber ($I_{exc} =$ 14.5kW/cm^2). No effects over time were observed, indicating that neither photophysical transitions, nor the absolute brightness of the dye was affected.

Parameters \ Buffer	AF647	IgG + AF647			DL755	IgG + DL755		
	PBS	PBS	GODCAT	GODCAT + 10 mM MEA	PBS	PBS	GODCAT	GODCAT + 10 mM MEA
k_{iso} (μs^{-1})	23.4	15.1	15.1	15.1	22.2	17	17	17
σ_{biso} (10^{-16} μs)	0.16	0.078	0.077	0.074	0.16	0.12	0.087	0.087
k_{iso}^{Th} (μs^{-1})	0.0011	0.0017	0.0005	0.005	0.01	0.01	0.005	0.007
k_{isc} (μs^{-1})	-	0.2	0.36	0.25	1.6	1.5	0.58	2.08
k_T (μs^{-1})	-	2.9	0.0008	3.08	0.16	0.26	0.001	0.17
k_{red} (μs^{-1})	0.011	0.24	0.0008	0.28	0.0058	0.0076	0.0002	0.61
k_{ox} (μs^{-1})	4.4×10^{-5}	1.9×10^{-5}	2.7×10^{-4}	1.7×10^{-4}	5.4×10^{-5}	1×10^{-5}	1×10^{-5}	7.3×10^{-5}
Q	0.01	0.21	0.21	0.21	0.09	0.2	0.17	0.17

Table S1. Fitted parameter values of photophysical transitions

Fitted parameter values from the TRAST experiments. Q values refer to the relative brightness of the P versus the N state (Eq. S13), as measured with the multiple notch filter for the 638 excitation and with a 770-850 nm filter for DL755 (750nm excitation).

A

Power series

	3%	5%	9%
Sx (median) (nm)	15.33 ± 3.99	14.01 ± 3.80	12.06 ± 2.86
Sy (median) (nm)	14.16 ± 3.81	13.07 ± 3.59	11.65 ± 2.91
EFO (Hz)	22156	33386	40408
Trace length (median) (a.u.)	15 ± 11.86	17.50 ± 15.57	29 ± 29.65
FGB (median) (Hz)	6897.15 ± 0	13794.29 ± 2556.43	17242.87 ± 2556.43
No. of vld locs (a.u.)	14194	48651	64860
No. of vld trace (a.u.)	1072	2912	2196

B

Dwell time series

	33 µs	166 µs	500 µs
Sx (median) (nm)	22.18 ± 9.53	13.34 ± 3.54	9.09 ± 1.86
Sy (median) (nm)	21.94 ± 9.30	12.59 ± 3.89	7.94 ± 1.65
EFO (Hz)	30258	33872	28583
Trace length (median) (a.u.)	8.0 ± 2.97	17.0 ± 14.83	36 ± 35.58
FGB (median) (Hz)	17245.10 ± 12783.80	15518.58 ± 2556.43	13793.10 ± 1704.14
No. of vld locs (a.u.)	51488	37556	52653
No. of vld trace (a.u.)	10876	2243	1029

C

Pattern repeat series

	Pattern repeat 1	Pattern repeat 5
Sx (median) (nm)	14.2 ± 3.99	13.93 ± 3.93
Sy (median) (nm)	13.3 ± 3.65	12.28 ± 3.49
EFO (Hz)	29810	30811
Trace length (median) (a.u.)	16.0 ± 13.34	16.00 ± 13.34
FGB (median) (Hz)	13794.29 ± 2556.43	13796.08 ± 2556.76
No. of vld locs (a.u.)	13995	23126
No. of vld trace (a.u.)	991	1374

Table S2. Imaging parameters obtained under different measurement conditions, related to Figure S22

Imaging parameters obtained for the NIR DNA PAINT MINFLUX images of NUP96 samples shown in Figure S22, recorded with different excitation powers (A), beam dwell times (B) and with or without a pattern repeat (C). The median standard deviation of localization in x and y direction (Sx & Sy), trace length, and frequence of background (FBG) were obtained from analyses of raw measurement data in pyMINFLUX (*45*). The reported values of effective frequency at offset (EFO) corresponds to the peak of the EFO histogram in pyMINFLUX. The number of valid localizations and valid traces were obtained directly from the raw data files.

Imaging 2D Seq	TCP parameter L	Minimum photon count	Dwell time (ms)	Pattern repeat	CFR (central frequency ratio)* limit	Laser power factor
Pre-localization	288	160	≥1	1	2	1
Iteration 1	288	150	≥1	5	Off	1
Iteration 2	151	100	≥1	5	0.8	2
Iteration 3	76	100	≥1	5	0.8	4
Iteration 4	40	150	≥1	5	2	6

Table S3. Standard Imaging 2D MINFLUX sequence

MINFLUX sequence used in simulations and imaging experiments. *: see (*34*) for a definition.

Figure	Imager concentration (pM)	Median localization precision (nm)		FRC resolution (nm)
		σ_x	σ_y	
Figure 6A	500	6.41 ± 2.30	5.42 ± 1.95	18.6 ± 0.2
Figure 6B	500	6.93 ± 2.7	5.21 ± 1.81	32.2 ± 0.7
Figure 6C	500	6.75 ± 2.14	5.58 ± 1.57	34.1 ± 0.2
Figure S21E	500	5.68 ± 1.74	5.09 ± 1.78	18.1 ± 0.8
Figure S21F	500	6.21 ± 2.22	5.20 ± 1.80	17.1 ± 0.2
Figure S21G	500	6.41 ± 2.30	5.42 ± 1.95	18.6 ± 0.2
Figure S21H	500	4.04 ± 1.72	3.33 ± 1.13	23.9 ± 0.3
Figure S23B	500	4.85 ± 1.88	4.14 ± 1.62	17.9 ± 0.8

Table S4. MINFLUX imaging parameters obtained in the imaging experiments
MINFLUX imaging parameters for the figures in main and supplementary texts.
The FRC analysis was done by pyMINFLUX v0.6.0 software (*45*) with temporal resolution of 600s and with 5 repeats.

SeqDefault_ 10pht	TCP parameter L	Minimum photon count	Dwell time (ms)	Pattern repeat	CFR (central frequency ratio)* limit	Laser power factor
Pre-localization	288	10	≥1	1	2	1
Iteration 1	288	10	≥1	5	0.5	1
Iteration 2	151	10	≥1	5	2	2
Iteration 3	101	10	≥1	5	0.8	4
Iteration 4	76	10	≥1	5	0.8	4
Iteration 5	40	10	≥1	5	2	6

Table S5. Custom MINFLUX sequence used in the imaging experiments reported in Fig S22
MINFLUX sequence in which the parameters pattern dwell times, pattern repeats and starting powers for the imaging experiments presented in Fig. S22.
